# High-Quality Genome-Scale Models From Error-Prone, Long-Read Assemblies

**DOI:** 10.3389/fmicb.2020.596626

**Published:** 2020-11-12

**Authors:** Jared T. Broddrick, Richard Szubin, Charles J. Norsigian, Jonathan M. Monk, Bernhard O. Palsson, Mary N. Parenteau

**Affiliations:** ^1^Exobiology Branch, Space Science and Astrobiology Division, NASA Ames Research Center, Moffett Field, CA, United States; ^2^Department of Bioengineering, University of California, San Diego, La Jolla, CA, United States

**Keywords:** antimicrobial resistance (AMR), metabolic model reconstruction, constraint-based model, nanopore sequencing, MinION nanopore device^®^, MinION long-read sequencing

## Abstract

Advances in nanopore-based sequencing techniques have enabled rapid characterization of genomes and transcriptomes. An emerging application of this sequencing technology is point-of-care characterization of pathogenic bacteria. However, genome assessments alone are unable to provide a complete understanding of the pathogenic phenotype. Genome-scale metabolic reconstruction and analysis is a bottom-up Systems Biology technique that has elucidated the phenotypic nuances of antimicrobial resistant (AMR) bacteria and other human pathogens. Combining these genome-scale models (GEMs) with point-of-care nanopore sequencing is a promising strategy for combating the emerging health challenge of AMR pathogens. However, the sequencing errors inherent to the nanopore technique may negatively affect the quality, and therefore the utility, of GEMs reconstructed from nanopore assemblies. Here we describe and validate a workflow for rapid construction of GEMs from nanopore (MinION) derived assemblies. Benchmarking the pipeline against a high-quality reference GEM of *Escherichia coli K*−12 resulted in nanopore-derived models that were >99% complete even at sequencing depths of less than 10× coverage. Applying the pipeline to clinical isolates of pathogenic bacteria resulted in strain-specific GEMs that identified canonical AMR genome content and enabled simulations of strain-specific microbial growth. Additionally, we show that treating the sequencing run as a mock metagenome did not degrade the quality of models derived from metagenome assemblies. Taken together, this study demonstrates that combining nanopore sequencing with GEM construction pipelines enables rapid, *in situ* characterization of microbial metabolism.

## Introduction

Recent advancements in sequencing technologies have opened up the possibility of *in situ* analysis of genomes and transcriptomes. In particular, the nanopore MinION sequencer (Oxford Nanopore Technologies, Oxford, United Kingdom) has emerged as a promising technology in this application space. The small size and computational footprint required to run the device (Lu et al., 2016) has enabled its use in human health settings, such as the detection of biothreat pathogens (Gargis et al., 2019), and in extreme environments such as Antarctica (Johnson et al., 2017) and the International Space Station (Castro-Wallace et al., 2017). The platform generates relatively long sequencing reads enabling the assembly of genomes at low coverage depth (Wick and Holt, 2019). This reduces the computational resources required to assemble genomes from nanopore reads, facilitating bioinformatic pipelines that can be run on a personal laptop (Castro-Wallace et al., 2017). However, despite advances in reagent chemistry, flowcell design and computational basecalling algorithms, the technology suffers from lower consensus genome accuracy compared with short-read sequencing techniques, especially in homopolymer regions (Gargis et al., 2019). Computational techniques exist to correct the frame-shift mutations that arise from these sequencing errors (Arumugam et al., 2019); however, resource intensive techniques may not be accessible in austere locales where the MinION device’s portability is a unique capability.

Point-of-care, rapid characterization of pathogenic bacteria is a promising *in situ* application of these technologies and techniques (van Belkum and Rochas, 2018; Monk, 2019). In standard hospital laboratories, short-read (Raven et al., 2019) and hybrid assembly (Hikichi et al., 2019) based methods successfully assessed methicillin-resistance in *Staphylococcus aureus* (hereafter *S. aureus*). Additionally, the nanopore sequencing technology rapidly profiled antimicrobial resistant (AMR) pathogens in a clinical setting along with characterization of patient microbiota (Leggett et al., 2020). However, a critique of genome-based approaches is their inability to elucidate an understanding of the pathogenic phenotype (Hendriksen et al., 2019). Recent developments in genome-scale metabolic modeling of pathogenic bacteria have shown promise in filling this gap between genotype and phenotype.

Genome-scale metabolic reconstruction is an emerging tool in the characterization and analysis of bacterial virulence and antibiotic resistance. These reconstructions are biochemical knowledge bases built from the annotated metabolic content encoded in the organism’s genome. When parameterized with constraints, these reconstructions become genome-scale models (GEMs) that simulate the organism’s phenotype under a given set of environmental conditions (O’Brien et al., 2015). To date, genome-scale reconstructions have been built for a wide range of pathogenic bacteria to include *Escherichia coli* (Monk et al., 2013), multiple strains of *Salmonella* (Seif et al., 2018), *Acinetobacter baumannii* (Norsigian et al., 2018), *S. aureus* (Seif et al., 2019), *Klebsiella pneumoniae* (Norsigian et al., 2019), and *Streptococcus oralis* (Jensen et al., 2020), among others. While the direct simulation of AMR mechanisms in these models is still in its infancy, the GEMs resulting from these reconstructions elucidated differential metabolic capabilities that provide a window into the phenotype of these pathogens beyond the simple presence-absence of AMR conferring genome content. For example, alternate nitrogen resource utilization in *Klebsiella pneumoniae* was used to classify strains by antibiotic resistance (Norsigian et al., 2019). Additionally, there is a growing consensus that bacterial metabolism represents fundamental constraints in the ability of bacteria to develop AMR evolutionary trajectories (Zampieri et al., 2017). As genome-scale metabolic reconstructions are based on an annotated genome, combining point-of-care sequencing with rapid metabolic model construction would augment existing genome-only assessments. However, it is unknown whether MinION assembly accuracy is sufficient for constructing high-quality genome-scale models.

## Materials and Methods

Additional methods detail and example code can be found in the Supplementary Material.

### Assembly and Reconstruction Pipeline

#### Read Filtering and Adapter Trimming

Details on MinION sequencing and basecalling are captured in the sample specific sections below. Basecalled reads in Fast5 format were concatenated into a single file using the command line. Multiplexed samples were separated by their respective barcodes using qcat^1^. Reads shorter than 1,000 bp (by default) were removed using Nanofilt (De Coster et al., 2018) and adapters were trimmed using Porechop^2^ with the –no_split argument, as implemented in the ONT bioinformatics package Pomoxis^3^.

#### Metagenome Size Determination

The filtered and trimmed reads were assembled using miniasm (Li, 2016) as implemented in Pomoxis with one round of Racon (Vaser et al., 2017) polishing to create a consensus sequence. This low quality assembly was parsed with SeqIO in the Biopython package (Cock et al., 2009) and the total genome/metagenome size was determined to be the sum of all sequences larger than 100 kbp.

#### Genome Assembly

The reads were assembled using Flye [v2.6, (Kolmogorov et al., 2019)]. The genome size parameter was determined above and the minimum overlap was set to 1,500 bp and the –plasmids argument was used. Additionally, for metagenomes, the –meta argument was included. For certain analyses the –asm_coverage argument was used and set to 70×. The resulting assembly was parsed and the total size was determined by summing all contigs with 10× or greater coverage. If the initial and final genome size parameters differed by a factor of 2 the assembly was repeated with the new genome size value.

#### Genome Polishing

The draft assembly from Flye was polished with two rounds of Racon after mapping the reads to the assembly using minimap2 (Li, 2016). The Racon polished assembly was polished with ONT’s Medaka consensus polishing tool (v0.9^4^).

#### Contig Binning

Contigs that were assessed to be circular based on the Flye assembler were separated from the linear contigs, as were contigs with less than 10× coverage. Linear contigs with GC content that different by less than 5%, determined by SeqIO, and coverage that differed by less than 15%, based on the Flye output, were grouped into a single folder for subsequent analysis.

#### Draft Genome-Scale Models Construction

All contigs were annotated with Prokka [v1.13 (Seemann, 2014)]. Draft genome-scale models were built by either taking the Genbank file output from Prokka as an input into a reference-based model building protocol (Norsigian et al., 2020b) or the protein sequences output from Prokka in Fasta format as an input into CarveMe (Machado et al., 2018). Content that was not included in the draft metabolic reconstructions was annotated by homology-based search using either the NCBI Blast search tools (Camacho et al., 2009) or DIAMOND search (Buchfink et al., 2015) against the Swissprot database downloaded from Uniprot [Downloaded: 2020/03/22, (The UniProt Consortium, 2019)].

#### Split ORF Recovery

Open reading frames that were split into multiple fragments due to sequencing errors were recovered by generating bidirectional (reciprocal) best-hit mappings between the protein sequences from Prokka and the Swissprot database. Adjacent genes with the same Swissprot best-hit were combined into a single amino acid sequence and output to a new file in the Fasta format. This new file was used in the reference-dependent protocol above.

### *Escherichia coli K*−12 str. BOP27 Validation Experiments

#### Culturing

*Escherichia coli K*−12 strain BOP27 from a frozen glycerol stock was first streaked on an LB agar plate and grown overnight at 37°C. Several mL of LB media was inoculated with a single colony and grown to late exponential phase.

#### DNA Extraction

The cells were pelleted by centrifugation and resuspended in 500 μL SETS buffer (75 mM NaCl, 25 mM EDTA pH 8, 20 mM Tris-HCl pH 7.5, 25% Sucrose). 5 μL RNaseA and 10 μL lysozyme were then added and the sample was incubated at 37°C for 60 min. 14 μL proteinase K and 30 μL 20% SDS were added, the sample was mixed gently by inversion and incubated at 55°C for 2 h inverting occasionally. 200 μL 5 M NaCl was added and the sample mixed thoroughly by gentle inversion. 500 μL chloroform was then added and the sample mixed by gentle inversion for 30 min at room temperature. Following centrifugation for 15 min at 4,500 × *g* at room temperature the upper aqueous phase was transferred to new 1.5 mL tube and another round of chloroform extraction was performed. The upper aqueous phase was transferred to new 1.5 mL tube. The volume was measured and 1/10 that volume of 3 M sodium acetate was added to the sample. DNA was precipitated with 0.7 volumes of isopropanol and the sample was placed on a slow rocker for 5 min. The filamentous genomic DNA precipitate was fished out with a Pasteur pipette, formed into a hook and sealed with a flame, and transferred to a series of 3 microcentrifuge tubes containing 1 mL 70% ethanol each. The final tube was centrifuged to pellet the DNA and the ethanol was removed with a pipette. The pellet was air dried for several minutes and resuspended in nuclease-free water. A Nanodrop was used to assess the quality of the genomic DNA prep, Qubit BR assay to check the concentration and Agilent TapeStation to check the size distribution.

#### MinION Sequencing

Native BOP27 genomic DNA (gDNA) was sequenced on a MinION R9.4 flowcell [Oxford Nanopore (ONT)]. The sequencing library was prepared using the ONT Rapid Barcoding Sequencing kit (SQK-RBK004) according to the manufacturer’s protocol with the following modifications: to two separate 0.2 mL PCR tubes, 1 and 0.5 μg gDNA, were diluted to 9 μL in ONT EB (10 mM Tris, 50 mM NaCl, pH 8.0). The barcoded fragmentation mix was added in a ratio of 3:1 and 1:1 (μg gDNA: μL fragmentation mix) to the 1 and 0.5 μg samples, respectively. Half the library (∼0.75 μg) was loaded onto the MinION flowcell without loading beads. ONT EB was used to bring the total library volume to 75 μL prior to loading. Sequencing was performed for 6 h on a flowcell with approximately 700 active pores. Sequencing Fast5 files were basecalled using the ONT Guppy basecaller (v3.2.2) with GPU acceleration on a laptop with an Intel i7-6550U processor and 8 GB RAM connected to an external GPU housing with an Nvidia GTX1070 (1920 CUDA cores, 8 GB VRAM) via a Thunderbolt 3 connection. Quality filtering was enabled with default settings using high accuracy, high accuracy with base pair modification, and fast base calling algorithms. Basecalled reads were demultiplexed with qcat (v1.1.0^5^) prior to assembly.

#### Assembly and Annotation

The pipeline above was used to generate draft assemblies and annotations for high accuracy, high accuracy with base pair modification, and fast basecalling methods. Subsets of reads at 15, 20, 40, and 100× coverage (before size filtering and adapter trimming) at N50 values of 20 and 10 k, were generated by randomly subsampling the total dataset (approximately 320× coverage). Additionally, a “worst-case” subset was generated by taking the 15× subset and removing all reads larger than 15 kbp. Overall accuracy of the BOP27 strain assembly versus the *E. coli* strain K12 substrain MG1655 reference was determined using the assess assembly function in Pomoxis^6^. Single nucleotide polymorphisms (SNPs) and InDels were determined using the dnadiff functions within MUMmer (Kurtz et al., 2004). The NanoFilt parameters were set to remove reads shorter than 2,000 base pairs prior to adapter and barcode trimming by Porechop. All assemblies were generated on a laptop computer equipped with an Intel i7-8650U processor and 16 GB of RAM.

#### Draft Model Construction

The pipeline above was used to generate draft genome scale metabolic reconstructions (GEM). The most recent *E. coli* strain K12 substrain MG1655 GEM, *i*ML1515 (Monk et al., 2017) was used as the reference model and reference genome (NCBI Reference Sequence: NC_000913.3). The Swissprot database downloaded from Uniprot [(The UniProt Consortium, 2019), downloaded: March 22, 2020] was used as the reference database and DIAMOND (Buchfink et al., 2015) was used to create the bidirectional best hit list. Model construction and simulation were performed in the Python programming language with COBRApy (Ebrahim et al., 2013) using the default GLPK solver in Jupyter Notebooks (Kluyver et al., 2016).

### Assembly and Reconstruction of Pathogenic Bacteria

#### Culturing Methods

Clinical isolates of *S. aureus*, *Acinetobacter baumannii* (hereafter: *A. baumannii*) and *Enterococcus faecium* (hereafter: *E. faecium*) were cultured in the same manner as described above for *E. coli.* DNA extraction leveraged the same protocol as that for *E. coli* above with the exception that lysostaphin was used instead of lysozyme.

#### MinION Sequencing

Native genomic DNA (gDNA) was sequenced on a MinION R9.4 flowcell [Oxford Nanopore (ONT)]. The sequencing library was prepared using the ONT Rapid Barcoding Sequencing kit (SQK-RBK004) according to the manufacturer’s protocol with the following modification: the gDNA input was increased to 800 ng genomic DNA and the optional SPRI bead cleanup was omitted. The run was allowed to proceed for approximately 6 h.

#### Assembly and Annotation

The pipeline above was used to generate draft assemblies and annotations for high accuracy (HAC) and high accuracy with base pair modification (HAC+mod) basecalling methods. The NanoFilt parameters were set to remove reads shorter than 1,000 base pairs prior to adapter and barcode trimming by Porechop. Flye was set to a minimum overlap of 1,500 bp and the –plasmid option was enabled. All assemblies were generated on a laptop computer equipped with an Intel i7-8650U processor and 16 GB of RAM.

#### Draft Model Construction

The pipeline above was used to generate draft genome scale metabolic reconstructions (GEM) from the draft genomes of the clinical isolates. For *S. aureus*, the most recent GEM of strain USA300 substrain TCH1516, *i*YS854 (Seif et al., 2019), was used as the reference model and reference genome (NCBI Reference Sequence: NC_010079.1). For *A. baumannii*, the most recent GEM of strain AYE, *i*CN718 (Norsigian et al., 2018), was used as the reference model and reference genome (NCBI Reference Sequence: NC_010410.1). The Swissprot database downloaded from Uniprot [(The UniProt Consortium, 2019), downloaded: March 22, 2020] was used as the reference database and DIAMOND (Buchfink et al., 2015) was used to create the bidirectional best hit list. Unique genome content was defined as putative open reading frames that did not map to the reference genome (percent ID cutoff of 80% and *e*-value cutoff of e-10). These proteins were annotated by homology search against the Swissprot database using DIAMOND. These annotations were manually curated for metabolic content, the putative biochemical reaction catalyzed determined from online databases [BiGG (Norsigian et al., 2020c) and KEGG (Kanehisa and Goto, 2000; Kanehisa et al., 2019)], and the reaction manually coded into the draft metabolic reconstruction to create complete, curated GEMs.

Draft metabolic reconstructions for the *E. faecium* clinical isolate were generated using a modified version of the multi-strain reconstruction pipeline described above (Supplementary Material) and the following reference genome-scale reconstructions: *Lactococcus lactis* subsp. cremoris MG1363, *i*NF516 (Flahaut et al., 2013), *E. coli* strain K12 substrain MG1655, *i*ML1515 (Monk et al., 2017), *Bacillus subtilis* strain 168, *i*YO844 (Oh et al., 2007), and *S. aureus* strain USA300 substrain TCH1516, *i*YS854 (Seif et al., 2019). A draft reconstruction was also generated using CarveMe (Machado et al., 2018) using the default settings. Model simulations and reconstruction were performed in the Python programming language with COBRApy (Ebrahim et al., 2013) using the default GLPK solver in Jupyter Notebooks (Kluyver et al., 2016).

#### Mock Metagenome Assembly

The metagenome was assembled following the pipeline without demultiplexing the barcoded reads prior to assembly. Bin contamination was manually determined by homology search of proteins annotated in each bin contig using NCBI Basic Local Alignment Search Tool [BLAST (Camacho et al., 2009)] against the non-redundant database.

#### Phylogenomic Analysis

Phylogenomic analysis was performed using GToTree (Lee, 2019) and the tools included therein (Edgar, 2004; Capella-Gutiérrez et al., 2009; Hyatt et al., 2010; Price et al., 2010; Eddy, 2011; Tange, 2018; Shen and Xiong, 2019). Phylogenomic analysis of the clinical isolates with other strains of the species was performed by taking the Prokka protein sequence FASTA file and comparing it to all Refseq assemblies for that species in the NCBI genome database. For placing the clinical isolates in the BiGG Models Database phylogeny, the database was parsed for the NCBI accession number used to reconstruct each GEM, the genomes were downloaded from NCBI and used for analysis. To place the BiGG models into a microbial tree of life, a table of all representative, complete archea and bacteria genomes was downloaded from the NCBI genome browser (approximately 3,200 genomes). From this list, a single representative from each phylum was selected (54 genomes) and the genbank file passed to GToTree for analysis. All phylogenomic trees were visualized using the Interactive Tree of Life web-based tool (Letunic and Bork, 2019). A list of NCBI accession numbers used can be found in the Supplementary Material.

## Results

### Assembly and Reconstruction Pipeline

**FIGURE 1:**
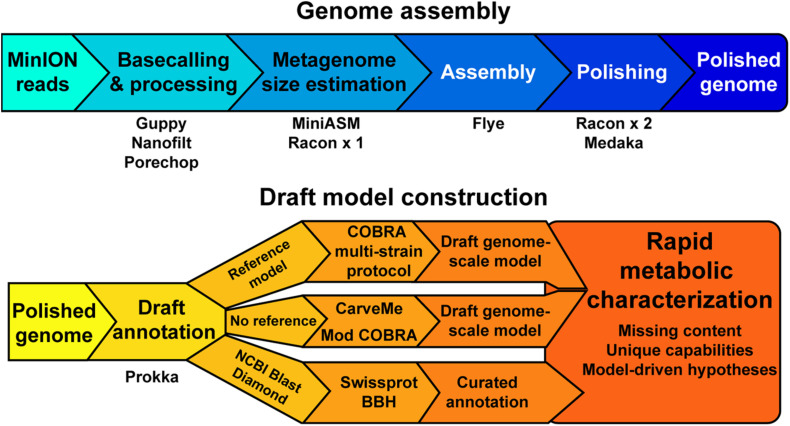
A schematic of the genome assembly and model construction pipeline used in this study.

Our objective was to assess the quality of genome-scale metabolic network reconstructions resulting from MinION-based assemblies. The assembly and draft reconstruction pipeline was designed to rapidly take extracted gDNA to a contextual framework for characterizing microbial metabolism (Figure 1). We leveraged existing studies that compare long-read assemblers to develop our assembly pipeline (Wick and Holt, 2019). The Flye assembler (Kolmogorov et al., 2019) showed the best balance of speed and accuracy and was used for all our assemblies. Flye requires an approximation of genome or metagenome size. Thus, we used a rapid, but inaccurate, assembly method [miniasm, (Li, 2016)] to approximate the genome size prior to assembly by Flye. This step can be bypassed if the approximate genome size is known. We polished the Flye assembly with multiple rounds of Racon (Vaser et al., 2017) before running the assembly polisher, Medaka, as recommended by ONT. We attempted to use existing binning tools for our metagenomes [BinSanity (Graham et al., 2017)]; however, these programs required computational resources in excess of a typical laptop. As our pipeline is designed to be used in austere field conditions, we implemented a simple binning strategy based on coverage and contig GC content. This approach requires manual curation of the binned contigs prior to annotation and model building.

We employed reference-dependent and independent strategies for generating draft metabolic reconstructions. We adapted a recently published protocol for building multi-strain metabolic reconstructions based on a reference model (Norsigian et al., 2020b). The input for this protocol requires a file in the NCBI Genbank format. The annotation tool Prokka includes a Genbank formatted output appropriate for this pipeline input. For organisms without a reference model of a closely related strain or species, we used CarveMe (Machado et al., 2018), a tool that uses the entire BiGG Models database (King et al., 2016) and homology search to generate a draft genome-scale network reconstruction. An additional automated reconstruction tool, modelSEED (Henry et al., 2010), is available but was not used as it performs similar or worse than CarveME (Machado et al., 2018) and it leverages a different name-space for reactions and metabolites, making model comparison difficult.

### Pipeline Validation With *E. coli K*−12

We assessed the ability of MinION sequencing and our pipeline to recapitulate the well-curated *E. coli* strain *K*−12 substrain MG1655 genome-scale model *i*ML1515 (Monk et al., 2017). A primary objective of this study was to optimize the trade-off in time versus accuracy; thus, we explored multiple basecalling strategies for the raw reads. CPU basecalling was prohibitively slow (on the order of days to weeks); thus, we only present GPU accelerated basecalling results, which was 100-times faster. The MinION generated approximately 200 k reads, 83% of which passed the *Q* score quality threshold for all basecaller methods; however, this 83% of the reads constituted 94% of the base pairs sequenced. The coverage depth for the *E. coli* genome varied depending on the basecalling method; the high accuracy algorithm (both with and without methylation calling) resulted in ∼307× coverage while the fast method resulted in only 260× coverage. The N50/N90 values were approximately 21/6 kbp for all methods. The high-accuracy basecalling model required 177 min while the fast method required 53 min (Table 1).

**TABLE 1:** Summary of assembly statistics for *E. coli* strain K12 substr. BOP27.

Basecaller	Coverage	Read N50 (bp)	Genome size (Mbp)	Contigs	*Q* score	SNPs	InDels	Basecalling time (min)	Assembly time (min)
HAC+	307×	22k	4.640	1	32.0	21	598	177	207
HAC−	306×	22k	4.639	1	27.3	4452	901	176	207
Fast	260×	22k	4.638	1	24.3	10621	4316	53	197

We compared the polished genomes with varying rounds of Racon combined with Medaka (Table 2). Overall, miniasm with one round of Racon was similar in accuracy to the Flye assembly with no additional polishing steps, although Flye alone resulted in a sixfold reduction in SNPs, likely due to Flye’s built in polishing step. Polishing the Flye assembly with Medaka increased the accuracy of the assembly compared to Flye alone. Two rounds of Racon were necessary and sufficient to maximize assembly accuracy (Table 2).

**TABLE 2:** Assembly quality for *E. coli* strain K12 substr. BOP27 at different steps in the pipeline for reads basecalled with the high accuracy algorithm with methylation calling enabled.

	Genome size (Mbp)	*Q*-score	SNPs	InDels
MiniASM_R1	4.636	25.6	470	9279
Flye	4.649	25.3	76	10482
Flye + Medaka	4.641	30.7	27	1660
Flye + R1 + M	4.639	30.9	32	654
Flye + R2 + M	4.640	32.0	21	598
Flye + R4 + M	4.640	32.0	25	596

**R#, Racon rounds; M, Medaka; SNP, single nucleotide polymorphism; InDel, insertions/deletions.**

We assessed the time versus accuracy tradeoff for three different basecalling models. The Guppy basecalling program can leverage a Fast algorithm as well as two high-accuracy (HAC) algorithms; one of which accounts for methylation of A and C nucleotides (HAC+mod). As previously mentioned, the HAC algorithm takes approximately 3 times longer than the Fast algorithm. The overall accuracy of the assemblies at similar coverage values (∼300× for both HAC algorithms and 260× for the Fast algorithm) were quite different with *Q* scores of 32.0, 27.3, and 24.3 for the HAC+mod, HAC, and Fast algorithms, respectively (Table 1). All three methods resulted in a single contig of a size that was shorter than the reference genome by 1.7 to 3.3 kbp. The HAC+mod algorithm reduced the number of SNPs by 200-fold compared to the HAC algorithm, suggesting DNA modification has a significant effect on read accuracy. The number of InDels in the HAC+mod algorithm was reduced by 33% compared to the HAC algorithm. The Fast algorithm resulted in an assembly with over 10,000 SNPs and 4,300 InDels (Table 1).

We evaluated the effect of coverage depth on the assembly accuracy and time for the basecalled reads. We subsampled the HAC+mod reads to generate two datasets with genome coverage of 15, 20, 40, and 100× coverage. The N50 value of one dataset was left at the original value (approximately 21 kbp) while one set was subsampled to have an N50 value of 11 kbp. These sets were then filtered to remove reads smaller than 2,000 bp and a quality score lower than 7, as well as adapter trimmed, resulting in final coverage values of 11, 14, 27, and 58×. An additional subset was generated from the 15× read set where all reads larger than 15 kbp were removed resulting in 7× coverage and an N50 of 9 kbp. We generated similar datasets for reads from the Fast algorithm.

**FIGURE 2:**
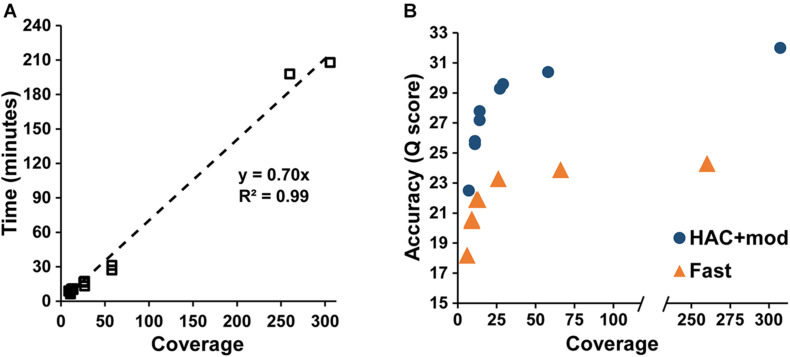
Assembly statistics versus coverage depth. **(A)** Assembly time versus genome coverage depth. **(B)** Assembly accuracy versus coverage depth.

Assembly accuracy increased rapidly with increasing coverage depth. For the HAC+mod datasets, all read subsets resulted in a single, circular contig except for the 7× coverage subset with all reads longer than 15 kbp removed (Supplementary Table 2). Assembly time increased linearly with coverage depth (Figure 2A); however, assembly accuracy increased non-linearly with the 58× coverage read set achieving a *Q* score of 30.4 compared to 32.0 for the highest coverage dataset (Figure 2B).

The Fast algorithm also resulted in single, circular contigs for most datasets. The exceptions were the worse-case scenario (6× coverage with all reads longer than 15 kbp removed), which resulted in 24 contigs, and a dataset with 9× coverage and an N50 of 22k, which resulted in 2 contigs. Similar to the HAC+mod algorithm, assembly accuracy increased non-linearly with a *Q* score of 23.9 at 66× coverage versus 24.3 at 260× (Figure 2B). For both algorithms, decreases in both SNPs and InDels followed the same non-linear trend (Supplementary Figures 1A,B). Based on these results, an assembly reaches 99% of the maximum accuracy at 80× coverage with an assembly time of 56 min for a genome size of approximately 5 Mbp.

**FIGURE 3:**
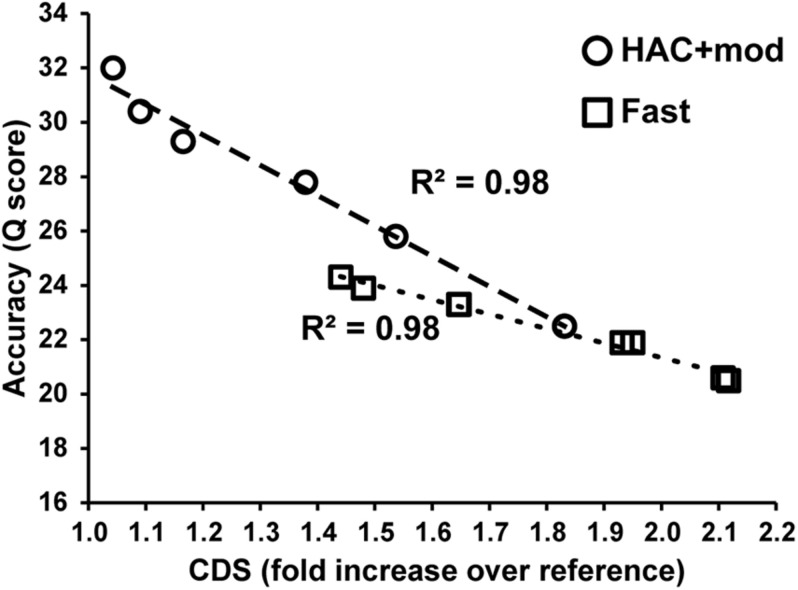
Assembly accuracy versus the number of coding DNA sequences annotated.

Errors in the assemblies affected the number of coding DNA sequence(s) (CDS) identified by Prokka. The *E. coli K*−12 MG1655 reference genome annotation contains 4305 CDS, 88 tRNA, and 22 rRNA. The HAC+mod assemblies had a higher number of CDS annotated that varied from a 4% to an 83% increase over the reference (Supplementary Table 2). The increase in the number of CDS was linearly correlated with assembly accuracy and can be used as a proxy for assembly quality (Figure 3). While the correlation was the same between HAC+mod and Fast algorithms (coefficient of determination = 0.98), the slope was different between the two algorithms due to the difference in maximum assembly accuracy.

### Genome-Scale Metabolic Reconstructions From MinION Assemblies

We used the outputs from the Prokka annotation to build genome-scale metabolic network reconstructions (GEMs) for three representative assemblies from the Fast and HAC+mod algorithms. We leveraged a recently published model building protocol that generates a reconstruction based on a reference model (Norsigian et al., 2020b). We added an additional step that attempts to recombine CDS that were split into multiple protein sequences due to sequencing errors. This homology-based correction resulted in a substantial recovery of split CDS. For the Fast basecalling algorithm, 76–84% of split CDS were recombined into a single protein sequence (Table 3). For the HAC+mod algorithm, this ranged from 82 to 96% with the highest coverage assembly achieving 4312 putative CDS versus 4305 for the reference *E. coli K*−12 MG1655 reference genome. We also leveraged an optional step in the process where the nucleotide sequences for each CDS in the reference genome is queried against the draft genome. Open reading frames missing from the draft annotation due to sequencing errors can be recovered using this step. A summary of recovered open reading frames is shown in Table 3.

**TABLE 3:** Statistics of genome-scale metabolic reconstructions built from the assembly and annotation pipeline.

Basecaller	Coverage	# contigs	Split CDS recovered (%)	ORFs recovered	Genes	Reactions	Growth rate (hr^–1^)
Fast	6×	24	4155 (76%)	547	1502	2712	0.877
	66×	1	1725 (84%)	40	1507	2712	0.877
	260×	1	1576 (83%)	32	1511	2712	0.877
HAC+mod	7×	5	2902 (82%)	233	1510	2710	0.877
	58×	1	360 (93%)	5	1515	2712	0.877
	307×	1	176 (96%)	3	1516	2712	0.877
iML1515	N/A	N/A	N/A	N/A	1516	2712	0.877

Using the most recent *E. coli K*−12 MG1655 GEM, *i*ML1515, as a reference model, we generated GEMs based on homology search between annotations of the corrected draft assemblies and the reference genome. The resulting models were surprisingly complete, even for the lowest accuracy assemblies (Table 3). When simulated, all models, even the lowest accuracy assembly from the Fast basecalling algorithm, predicted the same growth rate as the reference model. All metabolic reactions were present in the models, with the exception of the lowest accuracy HAC+mod assembly, which was missing 2 of 2,712 reactions. All models contained over 99% of the genes present in the reference model. Predicted growth capabilities on 298 possible carbon sources were identical for all six models and *i*ML1515 (Supplementary Figure 2).

### Assembly and Reconstruction of Pathogenic Bacteria

After validating the model generation pipeline on *E. coli K*−12, we applied the method to characterize clinical isolates of pathogenic bacteria. The clinical isolates included a hospital-acquired MRSA strain isolated from a patient with prolonged bacteremia secondary to osteomyelitis at Westchester Medical Center in Valhalla, New York. The *A. baumannii* strain was isolated from an osteomyelitis patient in 2017 in San Diego, CA, United States. The *E. faecium* strain was isolated from a patient in Cairo, Egypt. These isolates provided a practical application of the pipeline as antibiotic resistance genes can be identified by comparative analysis with reference models. Additionally, high-quality genome scale metabolic reconstructions exist for *A. baumannii* strain AYE (Norsigian et al., 2018) and *S. aureus* strain USA300 (Seif et al., 2019), which could serve as reference reconstructions. Currently a metabolic reconstruction is not available for *E. faecium*. Thus, we assessed the completeness of draft reconstructions of *E. faecium* using our pipeline compared to an automated reconstruction pipeline [CarveMe (Machado et al., 2018)]. The demultiplexed samples were basecalled, assembled and annotated using our pipeline, a summary of which is shown in Table 4. Circular contigs were generated for all three bacterial genomes. Additionally, plasmids were recovered for *A. baumannii* and *E. faecium.*

**TABLE 4:** Assembly statistics for clinical isolates of pathogenic bacteria characterized in this study.

				HAC+		HAC−		
					
Barcode	Species ID	Size (Mbp)	Cov.	ORFs	Mean ORF length (bp)	ORFs	Mean ORF length (bp)	Assembly time (min)
BC07	*A. baumannii*	3.93	13×	5826	561	5376	622	13
BC09	*E. faecium*	2.89	46×	3595	669	3217	762	34
BC11	*S. aureus*	2.84	75×	2961	803	2788	859	27

#### *Staphylococcus aureus* Clinical Isolate

The *S. aureus* isolate reads were assembled into a single circular contig that resulted in a near-complete genome-scale model. Overall genome coverage was approximately 75×, which, based off the pipeline validation in *E. coli*, was expected to result in a near-complete assembly. We generated assemblies using reads from both high accuracy algorithms, with and without accounting for methylation of A and C bases (HAC−mod and HAC+mod, respectively). The HAC−mod assembly resulted in fewer total ORFs with a longer mean length (Table 4). The reduction in fragmented ORFs suggested the HAC−mod algorithm resulted in a more accurate assembly than the HAC+mod algorithm, in contrast to the results in *E. coli* (Table 1).

The draft genome-scale model of the clinical isolate was nearly identical to that of the reference model. Using the pipeline’s split ORF recombination step reduced the number of predicted ORFs by 58 in the HAC−mod assembly and 145 in the HAC+mod; again suggesting the HAC−mod algorithm was more accurate due to fewer split ORFs. For both algorithms, only a single ORF was recovered by homology search of reference nucleotide sequences against the assemblies. The draft clinical isolate metabolic reconstruction consisted of 851 genes and 1,448 reactions compared to 866 genes and 1,455 reactions in the reference model *i*YS854. Of the 7 missing reactions none were essential and the growth rate predicted by the draft model, before curation, was identical to that of the reference model. Draft reconstructions were identical for both the HAC−mod and HAC+mod assemblies. Thus, we only curated the HAC−mod derived draft.

**FIGURE 4:**
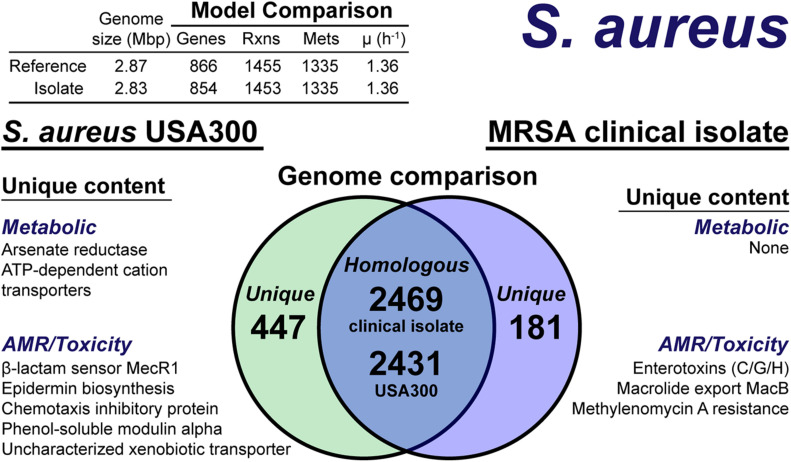
Comparison of *S. aureus* USA300 TCH1516 versus the *S. aureus* clinical isolate based on outputs of the genome-scale metabolic reconstruction pipeline.

The model construction pipeline provides an annotation of unique content, both in the reference strain and clinical isolate. Using these annotations, we rapidly curated the draft clinical isolate reconstruction into a complete model. The clinical isolate had 181 proteins without a clear homolog in the USA300 strain (Figure 4), of which 57 had homology to a protein the Swissprot database. From these 57 proteins, it was determined 16 were homologous to a protein in the reference, but below the 80% PID cutoff. Thus, we curated the final list of 41 proteins in the clinical isolate for unique metabolic and antibiotic resistance or toxicity content. A similar analysis of the reference strain USA300 TCH1516 resulted in 447 proteins without homology in the clinical isolate (Figure 4). Of these 477 proteins, 100 were homologous to Swissprot proteins and upon manual curation, we identified 67 as proteins of known function that were unique to the reference compared to the clinical isolate.

The metabolic capabilities of USA300 TCH1516 and the clinical isolate were almost identical. We first assessed the reactions missing from the draft model that were present in the reference. For 5 of the 7 missing reactions, we determined homology scores below the cutoff resulted in the reactions being removed from the draft reconstruction. However, after manual curation of these proteins against the Swissprot database homology search, we added the reactions back to the draft reconstruction of the clinical isolate. The final missing reactions were alternate peptidoglycan biosynthesis pathways in the reference strain. This alternate pathway is missing or different in the clinical isolate and its omission from the draft reconstruction was valid. The only unique capabilities in the reference strain genome were a putative arsenate reductase and a trio of poorly characterized ATP-dependent transporters with low homology to cation uptake proteins. There were no unique metabolic capabilities identified in the clinical isolate. The final, curated clinical isolate reconstruction, *i*SA854isolate (Supplementary Material), consisted of 854 genes, 1,453 reactions and 1,335 metabolites (Figure 4).

There were differences in the antibiotic resistance capabilities, host toxicity mechanisms and antimicrobial peptide biosynthesis between the reference strain and the clinical isolate (Figure 4). Of the methicillin resistance genes present in the reference strain, USA300 TCH1516, only the transmembrane β-lactam sensor, MecR1 (Peacock and Paterson, 2015), was missing in the clinical isolate. The clinical isolate genome encoded for a possible MacB efflux transporter (Kobayashi et al., 2001), which confers resistance to macrolides, such as erythromycin (Lin et al., 2009). The chemotaxis inhibitory protein, responsible for evading the host immune system (de Haas et al., 2004), and phenol-soluble modulins, capable of lysing host cells (Cheung et al., 2014), were not present in the clinical isolate. While both the reference strain and clinical isolate genomes encoded for Staphylococcal enterotoxin A, D, and E, the clinical isolate also encoded for enterotoxins C, G, and H. Biosynthesis of the antimicrobial lantibiotic, epidermin, was unique to the reference strain. No known lantibiotic biosynthesis pathways were present in the clinical isolate; however, a lantibiotic exporter was annotated. It should be noted, this analysis is based on genome comparison analysis and not direct outputs from model simulations as many of these mechanisms are out of scope for GEMs. However, the genome comparison was facilitated by the pipeline as only non-redundant content required manual intervention and annotation.

#### *Acinetobacter baumannii* Clinical Isolate

The *A. baumannii* isolate reads were assembled into one circular genome and one linear plasmid despite an overall genome coverage of approximately 13×. We generated assemblies using reads from both high accuracy algorithms, with and without accounting for methylation of A and G bases (HAC−mod and HAC+mod, respectively). Again, the HAC−mod assembly resulted in fewer total ORFs with a longer mean length (Table 4) and, thus, resulted in a more accurate assembly than the HAC+mod algorithm.

The metabolic content of the draft genome-scale model of the clinical isolate differed from that of the reference model. The pipeline’s split ORF recombination step reduced the number of predicted ORFs by 326 in the HAC−mod assembly suggesting the low genome coverage, and resulting accuracy, resulted in a substantial number of frameshifts. Five ORFs were recovered by homology search of reference nucleotide sequences against the assemblies. The draft clinical isolate metabolic reconstruction consisted of 675 genes and 1,007 reactions compared to 709 genes and 1,015 reactions in the reference model *i*CN718. Of the 8 missing reactions, one was essential; a capsular polysaccharide (CPS) biosynthetic enzyme, UDP-*N*-acetyl-D-glucosamine epimerase (model reaction UAG4E). Upon removing the product of this reaction, UDP-*N*-acetyl-D-galactosamine, from the CPS biomass reaction, the draft model was able to simulate growth. This observation, along with the absence of another CPS biosynthetic enzyme, UDP-*N*-acetyl-D-mannosamine oxidoreductase (model reaction UACMAMO), suggested differences in the CPS between the two species, which was evident during the subsequent manual curation phase.

**FIGURE 5:**
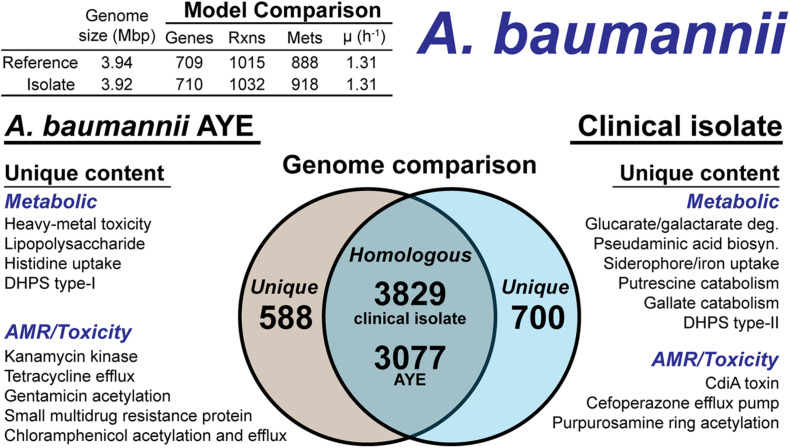
Comparison of *A. baumannii* AYE versus the *A. baumannii* clinical isolate based on outputs of the genome-scale metabolic reconstruction pipeline.

The *A. baumannii* clinical isolate had 700 proteins without a clear homolog in the AYE reference strain (Figure 5), of which 244 had homology to a protein the Swissprot database. From these 244 proteins, it was determined 83 were homologous to a protein in the reference, but below the 80% PID cutoff. Thus, we curated the final list of 161 proteins for unique metabolic and antibiotic resistance or toxicity content. A reciprocal analysis of the reference strain AYE resulted in 558 proteins without homology in the clinical isolate (Figure 5). Of these 558 proteins, 267 were homologous to Swissprot proteins and upon manual curation, we identified 179 as proteins of known function that were unique to the reference compared to the clinical isolate.

We first assessed the remaining reactions missing from the draft model that were present in the reference. Manual curation of the non-CPS missing reactions suggested these capabilities were indeed not present in the clinical isolate. These included acetolactate decarboxylase, proline racemase, allophanate hydrolase, an ABC-type histidine/cationic amino acid importer, and a plasmid encoded creatine amidohydrolase. Additionally, the DNA modifying enzyme cytosine-5-methyltransferase (model reaction CYTOM) was absent in the clinical isolate. Unique capabilities in the reference strain AYE genome included heavy metal efflux pumps for arsenic, and mercury as well as several genes related to CPS biosynthesis in addition to those mentioned above.

In contrast, there were several additional metabolic capabilities annotated in the clinical isolate. Degradation pathways for glucarate and galactarate, putrescine, and gallate were present in the isolate (Figure 5). These pathways were already present in the BiGG Models database (Norsigian et al., 2020c) in models for *E. coli* [*i*ML1515 (Monk et al., 2017)], *Bacillus subtilis* [*i*YO844 (Oh et al., 2007)], and *Pseudomonas putida* [*i*JN1462, (Nogales et al., 2020)]. As such, this new model content was easily transferred to the draft reconstruction and the gene-reaction associations updated with the clinical isolate ORFs (Supplementary Material). While both the clinical isolate and the reference strain contained a Type-I dihydropteroate synthase, the clinical isolate also encoded for a Type-II version of this enzyme. The Type-I is rapidly inhibited by sulfonaminde antibiotics while the Type-II may confer resistance to this class of antibiotics (Rådström et al., 1991).

The CPS biosynthesis loci in the clinical isolate suggested the presence of pseudaminic acid. The genetic organization of the clinical isolate CPS loci was highly similar to the pseudaminic acid containing K2 capsule (Kenyon et al., 2014). However, the K2 capsule is hypothesized to use *N*-acetyl-galactosamine as the initiation sugar (Kenyon et al., 2014). As mentioned above, the clinical isolate is missing the UDP-*N*-acetyl-D-glucosamine epimerase, which biosynthesizes *N*-acetyl-galactosamine. Thus, the initiating CPS sugar for the clinical isolate is unknown. The pseudaminic acid biosynthesis pathway was not in the BiGG Models Database. Thus, we manually curated the pathway into the draft reconstruction. There was one additional metabolic capability annotated in the clinical isolate but not added to the draft reconstruction. A gene cluster with very low homology to siderophore-mediated iron uptake was annotated. The low homology annotations prevented the content from being added to the model; however, this pathway is redundant with iron uptake systems present in both the reference strain and the clinical isolate. The final, curated clinical isolate reconstruction, *i*AB710isolate (Supplementary Material), consisted of 710 genes, 1,032 reactions and 918 metabolites (Figure 5).

There were differences in the antibiotic resistance capabilities and antimicrobial toxins between the *A. baumannii* reference strain and the clinical isolate (Figure 5). Genetic evidence for antibiotic resistance unique to the reference AYE strain included kanamycin, tetracycline, gentamicin, chloramphenicol and small multidrug resistance. The clinical isolate genome encoded for an ABC transporter that functions as a cefoperazone efflux pump (Yamanaka et al., 2016). Additionally, in the clinical isolate, there was evidence for an aminoglycoside acetyltransferase that acetylates aminoglycoside molecules conferring resistance to purpurosamine ring containing antibiotics (Nobuta et al., 1988). For antimicrobial toxins, the clinical isolate genome included a contact-dependent growth inhibition system, CdiA (Willett et al., 2015), which was not present in the reference genome. Efflux pumps and the aminoglycoside acetyltransferase are in-scope for the GEM and if combined with a model of antibiotic uptake kinetics could simulate AMR mechanisms. However, the CdiA mechanism is out of scope for GEMs and the analysis above is based on genome comparison.

#### *E. faecium* Draft Reconstruction

We attempted to generate a draft metabolic model of *Enterococcus faecium* (*E. faecium*) using a modified version of the pipeline. As a reference model does not exist for this species, we adapted the model construction pipeline to use a neighboring organism in the BiGG Models Database (Norsigian et al., 2020c) as a reference. As expected, phylogenomic analysis indicated the *E. faecium* strain clustered with the other Firmicutes in the BiGG database (Supplementary Figure 3). We also chose a “type-strain,” in this case *E. faecium* strain DO (NCBI: NC_017960.1), which was used to perform the ORF recovery step of the pipeline. Additionally, this version of the pipeline used an e-value threshold (1*e*-10) instead of PID to establish homology with the reference species. We generated draft metabolic reconstructions of *E. faecium* using three Firmicute GEMs due to their phylogeny as well as *E. coli* due to the model size and quality as reference species (Table 5).

**TABLE 5:** Draft metabolic reconstructions of *E. faecium* clinical isolate using different reference genome-scale reconstructions.

	Reference Strain	Ref model genes	Model Genes	Unique	No BBH hits
*E. faecium* GEM	*Lactococcus lactis*	517	342	35	1435
	*Bacillus subtilis*	844	319	21	1492
	*Staphylococcus aureus*	854	448	77	1545
	*Escherichia coli*	1515	434	97	1848

None of the *E. faecium* reconstructions created a complete model. The reconstructions varied in size from 319 to 448 genes (Table 5). However, each reconstruction also contained unique content not present in the draft models derived from the other reference species. The total, non-redundant draft reconstruction consisted of 626 genes, 1,045 reactions and 1,050 metabolites (Supplementary Material). This combined model was unable to simulate growth, likely due to the lack of a species-specific biomass reaction. The number of ORFs without homology to the reference species increased with increasing phylogenetic distance from *E. faecium* (Table 5). However, this did not correlate with model size as the most phylogenetically distance species, *E. coli*, resulted in the second largest model.

We compared the results from our pipeline with that from an automated reconstruction tool, CarveMe (Machado et al., 2018). CarveMe uses a universal model based on an older version of the BiGG database, which is conceptually similar to, but more comprehensive than, our use of multiple reference models above. CarveMe then removes model content based on a linear programming (MILP) approach. Annotated *E. faecium* protein sequences aligned to the CarveMe universal model protein database suggested 708 unique hits with an e-value above the threshold of 1*e*-10, which is similar to our combined model size of 626 genes. The CarveMe GEM contained 471 genes, 1,045 reactions and 784 metabolites. While this model was smaller than our combined model, it was capable of simulating growth on a chemically rich media (all exchange reactions open) while our combined model was not. The difference between the number of homologous proteins in our *E. faecium* assembly (708) and the final CarveMe model gene count (471) suggested the MILP “carving” process removed metabolic content that should likely be included in the *E. faecium* reconstruction. As the CarveMe method is a top-down model building tool, this is somewhat expected. There was unique content in both models. Of the 471 genes in the CarveMe model, 106 were missing from our combined model. At the same time, our combined model contained 261 genes not present in the CarveMe model. These results suggest the reconstructions resulting from both pipelines require manual curation.

#### Mock Metagenome

As analysis of microbial communities *in situ* is an important capability of the MinION sequencing platform, we assessed the quality of assemblies derived from treating the entire sequencing run as a mock metagenome. There were six separate sample preparations, or carry-over from previous runs, present in the mock metagenome including three strains of *E. coli*, with an order of magnitude difference in the read count between the least and most abundant sample (Supplementary Table 3). Single, circular contigs were recovered from the metagenome for *S. aureus*, *A. baumannii*, and *E. faecium*, similar in length to the assemblies from demultiplexed reads (Table 6). Comparison of the genomes derived from the HAC−mod reads and the metagenome resulted in *Q*-scores of 29.4, 34.2, and 43.6 for *A. baumannii*, *E. faecium*, and *S. aureus*, respectively (99.88% similar or better). Fragmented contigs for *Synechococcus elongatus* PCC 7942, assessed to be carry-over from a previous sequencing run on the same MinION flow cell, did not exceed the coverage cutoff for binning and annotation (10×). Reads for a substrain of *E. coli* CFT073 constituted 11% of the total reads but only 3% of the sequenced nucleotides (Supplementary Table 3) and did not exceed the coverage cutoff for binning. Surprisingly, the assembly for a substrain of *E. coli* O157:H7 was fragmented with highly variable coverage of the main chromosome (14–22×). This sample stood in stark contrast to the other assemblies above the cutoff threshold suggesting an issue during gDNA extraction or library construction. Still, the size of the assembled fragments was similar to the canonical size for this strain of *E. coli* (Table 6) and a draft reconstruction (not curated for novel content) based on *i*ML1515 was able to solve for growth (Supplementary Dataset).

**TABLE 6:** Assembly statistics for the MinION mock metagenome assembly.

Contigs	Contig ID	Mean coverage	# contigs	Mean GC content (%)	Size (bp)
Circular contigs	*A. baumannii*	16	1	39	3924200
	*A. baumannii* plasmid	125	1	38	19664
	*E. coli* O157:H7 Fragment	5	1	49	7257
	*E. coli* O157:H7 *Fragment*	23	1	55	2035
	*E. coli* O157:H7 Plasmid	50	1	48	92091
	*E. faecium*	57	1	38	2877951
	*E. faecium* plasmid	275	1	36	57023
	*E. faecium* plasmid	610	1	35	48025
	*E. faecium* plasmid	3164	1	39	2014
	*S. aureus*	80	1	33	2826253
	*S. elongatus* sp. PCC 7942	33	1	60	7842
Bin 1	*E. coli* O157:H7	17	3	50	737975
Bin 2	*E. coli* O157:H7	39	1	49	55052
Bin 3	*E. coli* O157:H7 plasmid	829	2	44	3300
Bin 4	*S. elongatus* sp. PCC 7942	12	1	53	43757
Bin 5	*E. coli* O157:H7	27	3*	50	4626033
Bin 6	*E. faecium* plasmid	71	1	36	164487

*2 kbp S. aureus contaminating contig.

As *A. baumannii* had the least similarity of the circular genomes, we ran the model reconstruction pipeline on the metagenome-derived assembly for this isolate. The metagenome-derived assembly contained 5,019 annotated ORFs with an average ORF length of 659 bp compared to 5,376 ORFs and 622 bp in the HAC−mod assembly. This result suggested assembly quality was higher for the metagenome than the demultiplexed assembly, possibly due to slightly higher coverage (Table 6). The draft reconstruction from the metagenome-derived assembly and the HAC−mod assembly were identical with 675 genes and 1,007 reactions, with the same 8 reference model reactions missing in both draft reconstructions. This result suggests near-complete genome scale metabolic reconstructions can be built with metagenome-derived assemblies.

## Discussion

Overall, the results from the assembly and model construction pipeline suggest accurate genome-scale metabolic reconstructions can be generated directly from MinION-based assemblies. The pipeline delivered >99% complete *E. coli* models for all coverage depths and assembly accuracies. Assembly accuracy rapidly increased with increasing coverage depth while the time required for assembly increased linearly with coverage depth (Figures 2A,B). These data suggest a coverage target of approximately 80× provides a balance between assembly accuracy and time required. At this value, an assembly is within 1% of its maximum accuracy and the pipeline can be complete in less than an hour on a laptop, for a genome of approximately 5 Mbp. While the Fast basecaller resulted in low accuracy, error prone assemblies, the reconstructions that resulted from these reads were still greater than 99% complete. This is likely due to the fact that network reconstructions require only a binary presence-absence assessment in order to add a reaction to the model. Taken together, this data suggests the GEM accuracy is equal to the genome assembly accuracy. As MinION assemblies achieve greater than 99% consensus accuracy, the resulting GEMs also achieve this level of completion. It should be noted that assembly quality and speed continue to increase due to advances in computational algorithms and hardware. For example, we validated the Supplementary Dataset example pipeline process on a laptop equipped with an intel i7-9750 45W processor with 32 GB RAM, and an RTX2060 GPU. High accuracy basecalling of the *E. coli* data took 90 min compared to 177 min (Guppy v. 3.6.1 vs. 3.2.2, mobile RTX2060 vs. desktop GTX1070) and assembly of a 40× HAC *E. coli* dataset took 12 min compared to 28 min on the i7-8650U processor.

The assembly pipeline resulted in circular contigs for *S. aureus*, *A. baumannii*, and *E. faecium* clinical isolates. The relationships between coverage depth and the overall genome accuracy and number of split ORFs were consistent with the validation results from *E. coli.* The recovery of the complete *A. baumannii* genome despite an overall coverage of 13×, was particularly encouraging. Additionally, the pipeline recovered plasmids for *A. baumannii* and *E. faecium*, an important capability as plasmids often contain AMR conferring genes (Buckner et al., 2018).

Treating the sequencing run as a metagenome did not degrade the quality of the resulting assemblies. In fact, our results for *A. baumannii* suggested an increase in assembly quality due to the incorporation of reads that are excluded from the demultiplexed samples due the lack of a barcode (approximately 11% of the reads, Supplementary Table 3). This result shows promise for rapid *in situ* characterization of simple microbial communities, for which there is precedence in the literature (Castro-Wallace et al., 2017; Arumugam et al., 2019; Sevim et al., 2019). The fragmented nature of the *E. coli* O157:H7 genome in the mock metagenome stood out from the quality assemblies of the other species. We hypothesized the presence of three different *E. coli* strains in the same metagenome may have affected the assembly quality. However, an assembly of the demultiplexed barcoded reads yielded similar results (Supplementary Table 4). Thus, the issue was inherent in the read data and the presence of multiple, similar strains did not affect the assembly quality. Consistency between the metabolic capabilities of the *S. aureus* clinical isolate and the reference strain, USA300 TCH1315, resulted in a solvable, near complete genome-scale model directly from the pipeline. The pairwise comparison output from the pipeline also enabled a rapid assessment of putative AMR capabilities (Figure 4). The observed differences, such as the presence of a putative MacB efflux pump in the clinical isolate, could assist in treatment selection or be used to correlate disease presentation and clinical outcomes with genomic content. These observations were based on comparative genomics and not a result of model simulations.

The metabolic capabilities of the *A. baumannii* clinical isolate diverged from the reference AYE strain (Figure 5). The presence of additional catabolic pathways, especially gallate, provide insight into the potential source and environmental background of the pathogen. Phylogenomic analysis of the clinical isolate identified *A. baumannii* strain XL380, isolated from cucumber rhizosphere, as the most similar (NCBI accession CP046536.1, Supplementary Figure 4). As gallate is a plant metabolite, it is plausible the clinical isolate also originated from a plant rhizosphere. Additionally, the pipeline generated sufficient detail about the clinical isolate’s capsular polysaccharide (CPS) to associate it with the K2 capsular (Kenyon et al., 2014), while at the same time deduced the initiating sugar is different in this strain. The presence of pseudaminic acid in the polysaccharide of *Helicobacter pylori* was correlated with increased virulence (Kao et al., 2016), again demonstrating the pipeline employed in this study has value in identifying metabolic content that may inform disease progression and likely clinical outcomes.

Manually curating additional content into the *A. baumannii* clinical isolate highlighted a potential bottleneck in the pipeline. The automated steps do allow for rapid identification of conserved content between a new strain and its corresponding reference, highlighting strains that may require significant manual curation. Still, the power of GEMs comes from the ability to simulate the metabolic phenotype. This requires the new content to be added to the *in silico* reconstruction. For content that is already present in the BiGG Models Database, this step is trivial and was accomplished in minutes (Supplementary Material). However, the manual curation of the pseudaminic acid biosynthesis pathway into the model did pose a barrier to rapidly simulating metabolic capabilities. The time investment is on the order of a few hours for manual curation of this pathway and its subsequent addition to the model. A solution to this challenge is to expand the number of pangenome-scale reconstructions. Pangenomes are compendia of all unique content in a given species (Norsigian et al., 2020a). For example, a pangenome of *A. baumannii* would have already included the pseudaminic acid biosynthesis pathway, as the K2 CPS containing species would have been included in the pangenome content. Pangenome-scale reconstructions require significant up-front effort as they require the curation of thousands of genes. Still, these reconstructions do exist in the BiGG database (Seif et al., 2018). Pangenome-scale reconstructions would be an important contribution to the implementation of point-of-care sequencing and metabolic characterization of AMR pathogens.

The primary limitation of our approach was evident in the attempt to reconstruct the *E. faecium* metabolic network. The lack of an *E. faecium* reference strain resulted in all reconstructions being approximately 50% complete. Using phylogeny to select the most appropriate reference strain appeared to minimize the number of protein sequences in *E. faecium* lacking homology to a protein in the reference strain (Table 5). However, it did not increase the completeness of the resulting reconstruction. This observation highlights the primary downside of our approach: the quality of the reference model is projected onto the new reconstruction. Combining multiple reference models resulted in a more complete reconstruction and mirrors the methodology of the CarveMe method (Machado et al., 2018). Still, the CarveMe-derived model did not include all metabolic content encoded in the *E. faecium* genome. This content may have been lost during the model reduction or “carving” step performed by this method. Additionally, the universal model used by CarveMe is based on an older version of the BiGG database that lacked two relevant GEMs: *i*YS854 for *S. aureus* USA300 TCH1315 and *i*NF514 for *L. lactis*, both of which are phylogenetic neighbors of *E. faecium* (Supplementary Figure 2). An update to the CarveMe universal model may result in a more complete reconstruction. For the draft resulting from our pipeline, manual curation is necessary to generate a complete reconstruction.

The primary objective of our investigation was to evaluate the quality of GEMs derived from MinION assemblies. The results from the *E. coli* assemblies and reconstructions suggest a GEM built from scratch using the MinION assembly from our pipeline would recapitulate the highly accurate *E. coli* model, *i*ML1515. Still, it is important to note we cannot assess the accuracy of the clinical isolate genomes and resulting GEMs as an assembly from an orthogonal, high-accuracy sequencing technology is not available, nor is there physiological data available to perform validation of GEM simulations. These constraints will likely be true in austere environments as well. Thus, while the MinION-derived *E. coli* GEMs and the growth rate simulations of the *S. aureus* and *A. baumannii* clinical isolate GEMs suggest near-complete reconstructions, it is important to highlight the lack of extensive accuracy and validation metrics.

Looking ahead, two growth areas are evident to increase the applicability of GEMs to point-of-care pathogen sequencing and characterization. First, directly simulating AMR mechanisms in GEMs would enable a quantitative assessment of the metabolic cost of antibiotic resistance and is an important next step. At the same time, it is important to note some AMR mechanisms are out of scope for metabolic modeling, including some identified in this study. For those mechanisms, there is no benefit of metabolic modeling over comparative genomics. Still, the results in this study indicate MinION-derived assemblies are of sufficient quality for these types of analysis.

**FIGURE 6:**
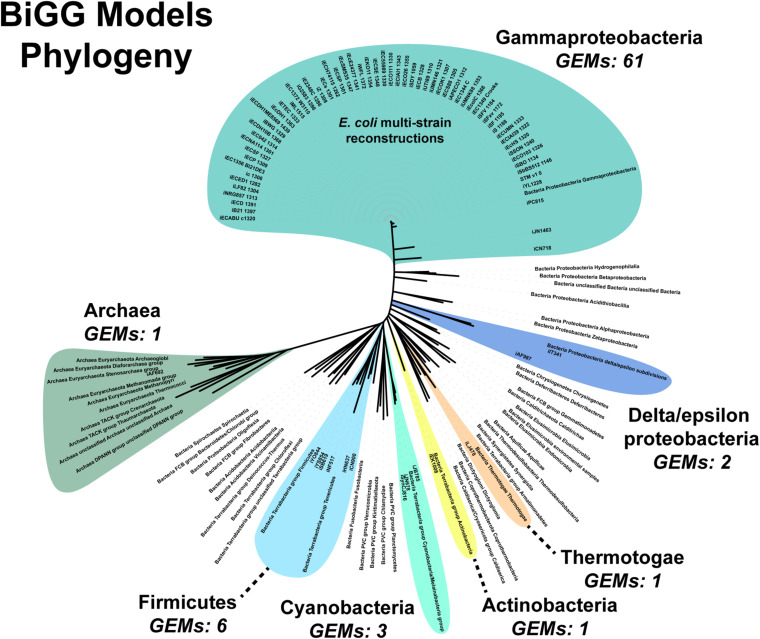
Phylogenomic analysis of genome scale models in the BiGG Models database.

An additional challenge is the requirement for manually curating the unique content of new strains. Fully automated pipelines, such as modelSEED, sacrifice some phenotypic prediction accuracy (Machado et al., 2018). Both our pipeline and the CarveMe method are reference-based reconstruction methods. Our pipeline uses a single reference model while CarveMe leverages the entire BiGG Models Database, a repository of high-quality, manually curated genome-scale reconstructions (Norsigian et al., 2020c). Still, the database’s microbial (archaea and prokaryote) model content is biased toward Gammaproteobacteria, which constitutes over 80% of the reconstructions present in the database (Figure 6). The next most abundant phylum is the Firmicutes at around 8%. Of the 54 bacterial and archaea phyla used in our analysis, genome-scale metabolic reconstructions are present in less than 15% (8/54 phyla). Additionally, as our results in *E. faecium* show, simply being in the same phylum does not result in a complete GEM, nor can automated reconstruction tools, such as CarveMe, completely bridge the gap between species. Thus, a significant expansion of manually-curated GEMs across the phylogenetic tree is needed. Our assembly and draft model construction pipeline can facilitate this expansion by minimizing the amount of manually curated unique content.

## Data Availability Statement

The datasets generated for this study can be found in the Supplementary Material. A subset of the *E. coli* MinION reads to run the assembly pipeline (28× coverage) as found in the Supplementary Material can be found at the SBRG Github (https://github.com/SBRG/MinIONtoModels/releases). Nanopore-derived genome assemblies of *E. coli* K-12 MG1655 BOP27 (300× coverage) and *A. baumannii*, *S. aureus*, and *E. faecium* clinical isolates (HAC-mod, demultiplexed) can be found at the National Center for Biotechnology Information BioProject Database under the ID PRJNA672694, BioSample accessions SAMN16574824, SAMN16574825, SAMN16574826, SAMN16574827.

## Author Contributions

JB and JM conceived and designed the study. CN and JB performed the computational analyses and simulations. RS and JB performed the culturing, gDNA extractions, and MinION sequencing. All authors discussed results and participated in the writing process.

## Conflict of Interest

The authors declare that the research was conducted in the absence of any commercial or financial relationships that could be construed as a potential conflict of interest.

## Footnotes

1 https://github.com/nanoporetech/qcat

2 https://github.com/rrwick/Porechop

3 https://github.com/nanoporetech/pomoxis

4 https://github.com/nanoporetech/medaka

5 https://github.com/nanoporetech/qcat

6 https://github.com/nanoporetech/pomoxis

## References

[B1] Arumugam K. Baǧcı C. Bessarab I. Beier S. Buchfink B. Górska A. (2019). Annotated bacterial chromosomes from frame-shift-corrected long-read metagenomic data. *Microbiome* 7:61. 10.1186/s40168-019-0665-y 30992083 PMC6469205

[B2] Buchfink B. Xie C. Huson D. H. (2015). Fast and sensitive protein alignment using DIAMOND. *Nat. Methods* 12 59–60. 10.1038/nmeth.3176 25402007

[B3] Buckner M. M. C. Ciusa M. L. Piddock L. J. V. (2018). Strategies to combat antimicrobial resistance: anti-plasmid and plasmid curing. *FEMS Microbiol. Rev.* 42 781–804. 10.1093/femsre/fuy031 30085063 PMC6199537

[B4] Camacho C. Coulouris G. Avagyan V. Ma N. Papadopoulos J. Bealer K. (2009). BLAST+: architecture and applications. *BMC Bioinformatics* 10:421. 10.1186/1471-2105-10-421 20003500 PMC2803857

[B5] Capella-Gutiérrez S. Silla-Martínez J. M. Gabaldón T. (2009). trimAl: a tool for automated alignment trimming in large-scale phylogenetic analyses. *Bioinformatics* 25 1972–1973. 10.1093/bioinformatics/btp348 19505945 PMC2712344

[B6] Castro-Wallace S. L. Chiu C. Y. John K. K. Stahl S. E. Rubins K. H. McIntyre A. B. R. (2017). Nanopore DNA sequencing and genome assembly on the international space station. *Sci. Rep.* 7:18022. 10.1038/s41598-017-18364-0 29269933 PMC5740133

[B7] Cheung G. Y. C. Joo H.-S. Chatterjee S. S. Otto M. (2014). Phenol-soluble modulins – critical determinants of staphylococcal virulence. *FEMS Microbiol. Rev.* 38 698–719. 10.1111/1574-6976.12057 24372362 PMC4072763

[B8] Cock P. J. A. Antao T. Chang J. T. Chapman B. A. Cox C. J. Dalke A. (2009). Biopython: freely available Python tools for computational molecular biology and bioinformatics. *Bioinformatics* 25 1422–1423. 10.1093/bioinformatics/btp163 19304878 PMC2682512

[B9] De Coster W. D’Hert S. Schultz D. T. Cruts M. Van Broeckhoven C. (2018). NanoPack: visualizing and processing long-read sequencing data. *Bioinformatics* 34 2666–2669. 10.1093/bioinformatics/bty149 29547981 PMC6061794

[B10] de Haas C. J. C. Veldkamp K. E. Peschel A. Weerkamp F. Van Wamel W. J. B. Heezius E. C. J. M. (2004). Chemotaxis inhibitory protein of *Staphylococcus aureus*, a bacterial antiinflammatory agent. *J. Exp. Med.* 199 687–695. 10.1084/jem.20031636 14993252 PMC2213298

[B11] Ebrahim A. Lerman J. A. Palsson B. O. Hyduke D. R. (2013). COBRApy: COnstraints-based reconstruction and analysis for python. *BMC Syst. Biol.* 7:74. 10.1186/1752-0509-7-74 23927696 PMC3751080

[B12] Eddy S. R. (2011). Accelerated profile HMM searches. *PLoS Comput. Biol.* 7:e1002195. 10.1371/journal.pcbi.1002195 22039361 PMC3197634

[B13] Edgar R. C. (2004). MUSCLE: multiple sequence alignment with high accuracy and high throughput. *Nucleic Acids Res.* 32 1792–1797. 10.1093/nar/gkh340 15034147 PMC390337

[B14] Flahaut N. A. L. Wiersma A. van de Bunt B. Martens D. E. Schaap P. J. Sijtsma L. (2013). Genome-scale metabolic model for *Lactococcus lactis* MG1363 and its application to the analysis of flavor formation. *Appl. Microbiol. Biotechnol.* 97 8729–8739. 10.1007/s00253-013-5140-2 23974365

[B15] Gargis A. S. Cherney B. Conley A. B. McLaughlin H. P. Sue D. (2019). Rapid detection of genetic engineering, structural variation, and antimicrobial resistance markers in bacterial biothreat pathogens by nanopore sequencing. *Sci. Rep.* 9 1–14. 10.1038/s41598-019-49700-1 31534162 PMC6751186

[B16] Graham E. D. Heidelberg J. F. Tully B. J. (2017). BinSanity: unsupervised clustering of environmental microbial assemblies using coverage and affinity propagation. *PeerJ* 5:e3035. 10.7717/peerj.3035 28289564 PMC5345454

[B17] Hendriksen R. S. Bortolaia V. Tate H. Tyson G. H. Aarestrup F. M. McDermott P. F. (2019). Using genomics to track global antimicrobial resistance. *Front. Public Health* 7:242. 10.3389/fpubh.2019.00242 31552211 PMC6737581

[B18] Henry C. S. DeJongh M. Best A. A. Frybarger P. M. Linsay B. Stevens R. L. (2010). High-throughput generation, optimization and analysis of genome-scale metabolic models. *Nat. Biotechnol.* 28 977–982. 10.1038/nbt.1672 20802497

[B19] Hikichi M. Nagao M. Murase K. Aikawa C. Nozawa T. Yoshida A. (2019). Complete genome sequences of eight methicillin-resistant *Staphylococcus aureus* strains isolated from patients in Japan. *Microbiol. Resour. Announc.* 8:e01212-19. 10.1128/MRA.01212-19 31753944 PMC6872886

[B20] Hyatt D. Chen G.-L. LoCascio P. F. Land M. L. Larimer F. W. Hauser L. J. (2010). Prodigal: prokaryotic gene recognition and translation initiation site identification. *BMC Bioinformatics* 11:119. 10.1186/1471-2105-11-119 20211023 PMC2848648

[B21] Jensen C. S. Norsigian C. J. Fang X. Nielsen X. C. Christensen J. J. Palsson B. O. (2020). Reconstruction and validation of a genome-scale metabolic model of *Streptococcus oralis* (iCJ415), a human commensal and opportunistic pathogen. *Front. Genet.* 11:116. 10.3389/fgene.2020.00116 32194617 PMC7063969

[B22] Johnson S. S. Zaikova E. Goerlitz D. S. Bai Y. Tighe S. W. (2017). Real-time DNA sequencing in the antarctic dry valleys using the oxford nanopore sequencer. *J. Biomol. Tech.* 28 2–7. 10.7171/jbt.17-2801-009 28337073 PMC5362188

[B23] Kanehisa M. Goto S. (2000). KEGG: Kyoto encyclopedia of genes and genomes. *Nucleic Acids Res.* 28 27–30. 10592173 10.1093/nar/28.1.27 PMC102409

[B24] Kanehisa M. Sato Y. Furumichi M. Morishima K. Tanabe M. (2019). New approach for understanding genome variations in KEGG. *Nucleic Acids Res.* 47 D590–D595. 10.1093/nar/gky962 30321428 PMC6324070

[B25] Kao C.-Y. Sheu B.-S. Wu J.-J. (2016). *Helicobacter* pylori infection: an overview of bacterial virulence factors and pathogenesis. *Biomed. J.* 39 14–23. 10.1016/j.bj.2015.06.002 27105595 PMC6138426

[B26] Kenyon J. J. Marzaioli A. M. Hall R. M. De Castro C. (2014). Structure of the K2 capsule associated with the KL2 gene cluster of *Acinetobacter baumannii*. *Glycobiology* 24 554–563. 10.1093/glycob/cwu024 24688093

[B27] King Z. A. Lu J. Dräger A. Miller P. Federowicz S. Lerman J. A. (2016). BiGG Models: a platform for integrating, standardizing and sharing genome-scale models. *Nucleic Acids Res.* 44 D515–D522. 10.1093/nar/gkv1049 26476456 PMC4702785

[B28] Kluyver T. Ragan-Kelley B. Pérez F. Granger B. Bussonnier M. Frederic J. (2016). “Jupyter notebooks – a publishing format for reproducible computational workflows,” in *Positioning and Power in Academic Publishing: Players, Agents and Agendas*, eds Loizides F. Schmidt B. (Amsterdam: IOS Press), 87–90.

[B29] Kobayashi N. Nishino K. Yamaguchi A. (2001). Novel macrolide-specific ABC-type efflux transporter in *Escherichia coli*. *J. Bacteriol.* 183 5639–5644. 10.1128/JB.183.19.5639-5644.2001 11544226 PMC95455

[B30] Kolmogorov M. Yuan J. Lin Y. Pevzner P. A. (2019). Assembly of long, error-prone reads using repeat graphs. *Nat. Biotechnol.* 37 540–546. 10.1038/s41587-019-0072-8 30936562

[B31] Kurtz S. Phillippy A. Delcher A. L. Smoot M. Shumway M. Antonescu C. (2004). Versatile and open software for comparing large genomes. *Genome Biol.* 9:R12. 10.1186/gb-2004-5-2-r12 PMC395750 14759262

[B32] Lee M. D. (2019). GToTree: a user-friendly workflow for phylogenomics. *Bioinformatics* 35 4162–4164. 10.1093/bioinformatics/btz188 30865266 PMC6792077

[B33] Leggett R. M. Alcon-Giner C. Heavens D. Caim S. Brook T. C. Kujawska M. (2020). Rapid MinION profiling of preterm microbiota and antimicrobial-resistant pathogens. *Nat. Microbiol.* 5 430–442. 10.1038/s41564-019-0626-z 31844297 PMC7044117

[B34] Letunic I. Bork P. (2019). Interactive tree of life (iTOL) v4: recent updates and new developments. *Nucleic Acids Res.* 47 W256–W259. 10.1093/nar/gkz239 30931475 PMC6602468

[B35] Li H. (2016). Minimap and miniasm: fast mapping and de novo assembly for noisy long sequences. *Bioinformatics* 32 2103–2110. 10.1093/bioinformatics/btw152 27153593 PMC4937194

[B36] Lin H. T. Bavro V. N. Barrera N. P. Frankish H. M. Velamakanni S. van Veen H. W. (2009). MacB ABC transporter is a dimer whose ATPase activity and macrolide-binding capacity are regulated by the membrane fusion protein MacA. *J. Biol. Chem.* 284 1145–1154. 10.1074/jbc.M806964200 18955484 PMC2613632

[B37] Lu H. Giordano F. Ning Z. (2016). Oxford nanopore MinION sequencing and genome assembly. *Genomics Proteomics Bioinformatics* 14 265–279. 10.1016/j.gpb.2016.05.004 27646134 PMC5093776

[B38] Machado D. Andrejev S. Tramontano M. Patil K. R. (2018). Fast automated reconstruction of genome-scale metabolic models for microbial species and communities. *Nucleic Acids Res.* 46 7542–7553. 10.1093/nar/gky537 30192979 PMC6125623

[B39] Monk J. M. (2019). Predicting antimicrobial resistance and associated genomic features from whole-genome sequencing. *J. Clin. Microbiol.* 57:e01610-18. 10.1128/JCM.01610-18 30463894 PMC6355531

[B40] Monk J. M. Charusanti P. Aziz R. K. Lerman J. A. Premyodhin N. Orth J. D. (2013). Genome-scale metabolic reconstructions of multiple *Escherichia coli* strains highlight strain-specific adaptations to nutritional environments. *Proc. Natl. Acad. Sci. U.S.A.* 110 20338–20343. 10.1073/pnas.1307797110 24277855 PMC3864276

[B41] Monk J. M. Lloyd C. J. Brunk E. Mih N. Sastry A. King Z. (2017). iML1515, a knowledgebase that computes *Escherichia coli* traits. *Nat. Biotechnol.* 35 904–908. 10.1038/nbt.3956 29020004 PMC6521705

[B42] Nobuta K. Tolmasky M. E. Crosa L. M. Crosa J. H. (1988). Sequencing and expression of the 6’-N-acetyltransferase gene of transposon Tn1331 from *Klebsiella pneumoniae*. *J. Bacteriol.* 170 3769–3773. 10.1128/jb.170.8.3769-3773.1988 2841303 PMC211361

[B43] Nogales J. Mueller J. Gudmundsson S. Canalejo F. J. Duque E. Monk J. (2020). High-quality genome-scale metabolic modelling of *Pseudomonas* putida highlights its broad metabolic capabilities. *Environ. Microbiol.* 22 255–269. 10.1111/1462-2920.14843 31657101 PMC7078882

[B44] Norsigian C. J. Attia H. Szubin R. Yassin A. S. Palsson B. Ø Aziz R. K. (2019). Comparative genome-scale metabolic modeling of metallo-beta-lactamase–producing multidrug-resistant *Klebsiella pneumoniae* clinical Isolates. *Front. Cell Infect. Microbiol.* 9:161. 10.3389/fcimb.2019.00161 31179245 PMC6543805

[B45] Norsigian C. J. Fang X. Palsson B. O. Monk J. M. (2020a). “Pangenome flux balance analysis toward panphenomes,” in *The Pangenome: Diversity, Dynamics and Evolution of Genomes*, eds Tettelin H. Medini D. (Cham: Springer International Publishing), 219–232. 10.1007/978-3-030-38281-0_10 32633918

[B46] Norsigian C. J. Fang X. Seif Y. Monk J. M. Palsson B. O. (2020b). A workflow for generating multi-strain genome-scale metabolic models of prokaryotes. *Nat. Protoc.* 15 1–14. 10.1038/s41596-019-0254-3 31863076 PMC7017905

[B47] Norsigian C. J. Pusarla N. McConn J. L. Yurkovich J. T. Dräger A. Palsson B. O. (2020c). BiGG Models 2020: multi-strain genome-scale models and expansion across the phylogenetic tree. *Nucleic Acids Res.* 48 D402–D406. 10.1093/nar/gkz1054 31696234 PMC7145653

[B48] Norsigian C. J. Kavvas E. Seif Y. Palsson B. O. Monk J. M. (2018). iCN718, an Updated and improved genome-scale metabolic network reconstruction of *Acinetobacter baumannii* AYE. *Front. Genet.* 9:121. 10.3389/fgene.2018.00121 29692801 PMC5902709

[B49] O’Brien E. J. Monk J. M. Palsson B. O. (2015). Using genome-scale models to predict biological capabilities. *Cell* 161 971–987. 10.1016/j.cell.2015.05.019 26000478 PMC4451052

[B50] Oh Y.-K. Palsson B. O. Park S. M. Schilling C. H. Mahadevan R. (2007). Genome-scale reconstruction of metabolic network in bacillus subtilis based on high-throughput phenotyping and gene essentiality data. *J. Biol. Chem.* 282 28791–28799. 10.1074/jbc.M703759200 17573341

[B51] Peacock S. J. Paterson G. K. (2015). Mechanisms of methicillin resistance in *Staphylococcus aureus*. *Annu. Rev. Biochem.* 84 577–601. 10.1146/annurev-biochem-060614-034516 26034890

[B52] Price M. N. Dehal P. S. Arkin A. P. (2010). FastTree 2 – approximately maximum-likelihood trees for large alignments. *PLoS One* 5:e9490. 10.1371/journal.pone.0009490 20224823 PMC2835736

[B53] Rådström P. Swedberg G. Sköld O. (1991). Genetic analyses of sulfonamide resistance and its dissemination in gram-negative bacteria illustrate new aspects of R plasmid evolution. *Antimicrob. Agents Chemother.* 35 1840–1848. 10.1128/aac.35.9.1840 1952855 PMC245278

[B54] Raven K. E. Blane B. Leek D. Churcher C. Kokko-Gonzales P. Pugazhendhi D. (2019). Methodology for whole-genome sequencing of methicillin-resistant *Staphylococcus aureus* Isolates in a routine hospital microbiology laboratory. *J. Clin. Microbiol.* 57:e00180-19. 10.1128/JCM.00180-19 30894439 PMC6535593

[B55] Seemann T. (2014). Prokka: rapid prokaryotic genome annotation. *Bioinformatics* 30 2068–2069. 10.1093/bioinformatics/btu153 24642063

[B56] Seif Y. Kavvas E. Lachance J.-C. Yurkovich J. T. Nuccio S.-P. Fang X. (2018). Genome-scale metabolic reconstructions of multiple *Salmonella* strains reveal serovar-specific metabolic traits. *Nat. Commun.* 9:3771. 10.1038/s41467-018-06112-5 30218022 PMC6138749

[B57] Seif Y. Monk J. M. Mih N. Tsunemoto H. Poudel S. Zuniga C. (2019). A computational knowledge-base elucidates the response of *Staphylococcus aureus* to different media types. *PLoS Comput. Biol.* 15:e1006644. 10.1371/journal.pcbi.1006644 30625152 PMC6326480

[B58] Sevim V. Lee J. Egan R. Clum A. Hundley H. Lee J. (2019). Shotgun metagenome data of a defined mock community using Oxford Nanopore. PacBio and Illumina technologies. *Sci. Data* 6 1–9. 10.1038/s41597-019-0287-z 31772173 PMC6879543

[B59] Shen W. Xiong J. (2019). TaxonKit: a cross-platform and efficient NCBI taxonomy toolkit. *Bioinformatics* [Preprint]. 10.1101/513523

[B60] Tange O. (2018). *GNU Parallel 2018.* Frederiksberg: Ole Tange 10.5281/zenodo.1146014

[B61] The UniProt Consortium. (2019). UniProt: a worldwide hub of protein knowledge. *Nucleic Acids Res.* 47 D506–D515. 10.1093/nar/gky1049 30395287 PMC6323992

[B62] van Belkum A. Rochas O. (2018). Laboratory-based and point-of-care testing for MSSA/MRSA detection in the age of whole genome sequencing. *Front. Microbiol.* 9:1437. 10.3389/fmicb.2018.01437 30008711 PMC6034072

[B63] Vaser R. Sovic I. Nagarajan N. Sikic M. (2017). Fast and accurate de novo genome assembly from long uncorrected reads. *Genome Res*. 27, 737–746. 28100585 10.1101/gr.214270.116 PMC5411768

[B64] Wick R. R. Holt K. E. (2019). Benchmarking of long-read assemblers for prokaryote whole genome sequencing. *F1000Res.* 8:2138 10.12688/f1000research.21782.1 PMC6966772 31984131

[B65] Willett J. L. E. Gucinski G. C. Fatherree J. P. Low D. A. Hayes C. S. (2015). Contact-dependent growth inhibition toxins exploit multiple independent cell-entry pathways. *PNAS* 112 11341–11346. 10.1073/pnas.1512124112 26305955 PMC4568652

[B66] Yamanaka Y. Shimada T. Yamamoto K. Ishihama A. (2016). Transcription factor CecR (YbiH) regulates a set of genes affecting the sensitivity of *Escherichia coli* against cefoperazone and chloramphenicol. *Microbiology* 162 1253–1264. 10.1099/mic.0.000292 27112147

[B67] Zampieri M. Enke T. Chubukov V. Ricci V. Piddock L. Sauer U. (2017). Metabolic constraints on the evolution of antibiotic resistance. *Mol. Syst. Biol.* 13:917. 10.15252/msb.20167028 28265005 PMC5371735

