# Supplementary material for: High-Quality Genome-Scale Models From Error-Prone, Long-Read Assemblies
Source: Front Microbiol. 2020 Nov 12;11:596626. doi: 10.3389/fmicb.2020.596626 (PMC7688782; doi:10.3389/fmicb.2020.596626)
Supplement: Supplementary file 1 [file Data_Sheet_1.ZIP › Supplemental Materials/Supplementary_Material_Broddrick_Models_from_MinION.docx]

Supplementary Material

**High-quality genome-scale models from error-prone long-read assemblies**

Jared T. Broddrick, Richard Szubin, Charles J. Norsigian, Jonathan M. Monk, Bernhard O. Palsson and Mary N. Parenteau

# Supplementary Materials and Methods

The code used for all steps of the pipeline are found in the Supplementary Data Files.

**Basecalling**:

*Guppy basecalling commands:*

GTX1070 eGPU, Guppy v3.2.2

guppy_basecaller --input_path XXX --save_path XXX -c dna_r9.4.1_450bps_modbases_dam-dcm-cpg_hac.cfg -r --qscore_filtering -q 0 -x 'cuda:0' --gpu_runners_per_device 3 --chunks_per_runner 1024

ONT Guppy basecalling software version 3.2.2+9fe0a78

config file: /opt/ont/guppy/data/dna_r9.4.1_450bps_modbases_dam-dcm-cpg_hac.cfg

model file: /opt/ont/guppy/data/template_r9.4.1_450bps_modbases_dam-dcm-cpg_hac.jsn

input path:         XXX/fast5

save path:          XXX

chunk size:         1000

chunks per runner:  1024

minimum qscore:     7

records per file:   0

num basecallers:    4

gpu device:         cuda:0

kernel path:

runners per device: 3

Found 51 fast5 files to process.

Init time: 2276 ms

Caller time: 10600198 ms, Samples called: 21764824214, samples/s: 2.05325e+06

Finishing up any open output files.

Basecalling completed successfully.

RTX2060 Mobile, Guppy v3.6.1

guppy_basecaller --input_path XXX --save_path XXX -c dna_r9.4.1_450bps_modbases_dam-dcm-cpg_hac.cfg -r --qscore_filtering -q 0 -x 'cuda:0' --gpu_runners_per_device 8 --chunks_per_runner 2560 --num_callers 6

ONT Guppy basecalling software version 3.6.1+249406c, client-server API version 1.1.0

config file:        /ont-guppy/data/dna_r9.4.1_450bps_modbases_dam-dcm-cpg_hac.cfg

model file:         /ont-guppy/data/template_r9.4.1_450bps_modbases_dam-dcm-cpg_hac.jsn

input path:         XXX/fast5

save path:          XXX

chunk size:         2000

chunks per runner:  2560

minimum qscore:     7

records per file:   0

num basecallers:    6

gpu device:         cuda:0

kernel path:

runners per device: 8

Found 51 fast5 files to process.

Init time: 1313 ms

Caller time: 5583001 ms, Samples called: 21764824214, samples/s: 3.89841e+06

Finishing up any open output files.

Basecalling completed successfully.

**Manual curation of Genome-Scale Network Reconstructions:**

*Transfer of metabolic reactions between genome-scale reconstructions:* Based on homology, the metabolic reactions for glucarate and galactarate, putrescine, and gallate degradation present in the *A. baumannii* clinical isolate genome were annotated. The biochemical reactions for each step in the pathway was compared to existing content in the BiGG database (King et al., 2016; Norsigian et al., 2020). For each reaction, the source GEM and model reaction ID were recorded. The source GEMs were downloaded from BiGG and each reaction was copied from the source model to the clinical isolate model and the gene-reaction association was updated to the clinical isolate ORF using Cobrapy (Ebrahim et al., 2013) using the following Python pseudocode:

source_model = cobra.io.load_json_model(‘model.json’)

source_rxns = {‘rxn_id’:’new_gene_id’}

for k,v in source_rxns.items():

rxn = source_model.reactions.get_by_id(k)

for mets in rxn.metabolites:

if mets not in model.metabolites:

m = mets.copy()

model.add_metabolites(m)

rxn.gene_reaction_rule = v

new_model.add_reaction(rxn)

# Supplementary Data

All genome-scale metabolic network reconstructions developed for this work can be found in the Supplementary Data Files.

- *Ecoli_O157_draft.json*: Uncurated reconstruction of *E. coli* O157:H7
- *iAB710isolate.json*: Curated reconstruction of the *A. baumannii* clinical isolate in this investigation.
- *iEF_CarveMe.json*: Uncurated, CarveMe-derived model of the *E. faecium* clinical isolate in this investigation.
- *iEF_combo_COBRA_pipeline.json*: Uncurated reconstruction of the *E. faecium* clinical isolate in this investigation based on aggregation of content derived from the multi-strain pipeline using iML1515, iYS854, *i*NF514 and *i*YO844 as reference GEMs (see Main Text).
- *iAB710isolate.json*: Curated reconstruction of the *A. baumannii* clinical isolate in this investigation.

# Supplementary Figures and Tables

## Supplementary Figures


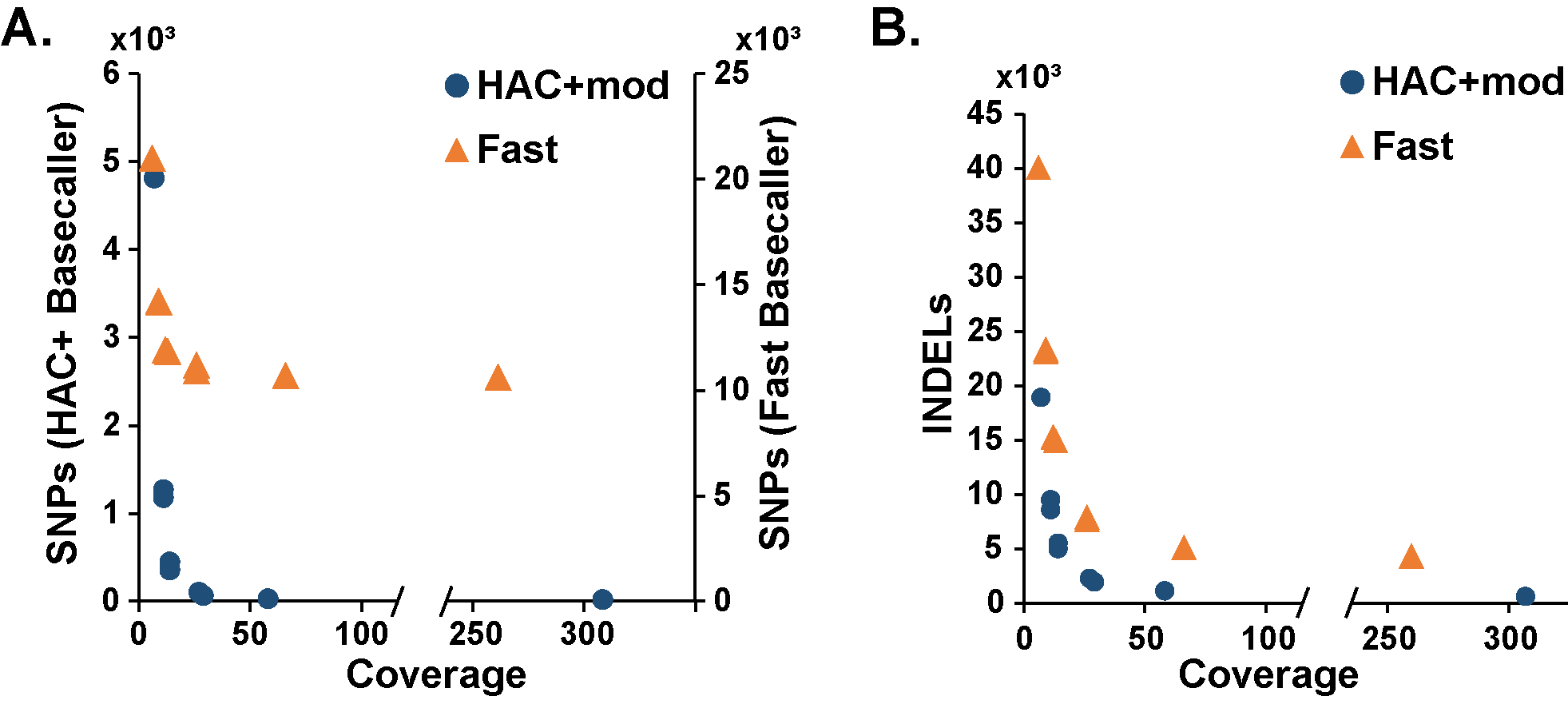


**Supplementary Figure 1.** Sequencing errors as a function of sequencing coverage. A. Number of single-nucleotide polymorphisms (SNPs) in the assemblies based on sequencing reads from the high accuracy + methylation calling algorithm (blue circles) and the fast basecalling algorithm (orange triangles). B. Number of insertion/deletions (InDels) in the assemblies based on sequencing reads from the high accuracy + methylation calling algorithm (blue circles) and the fast basecalling algorithm (orange triangles).


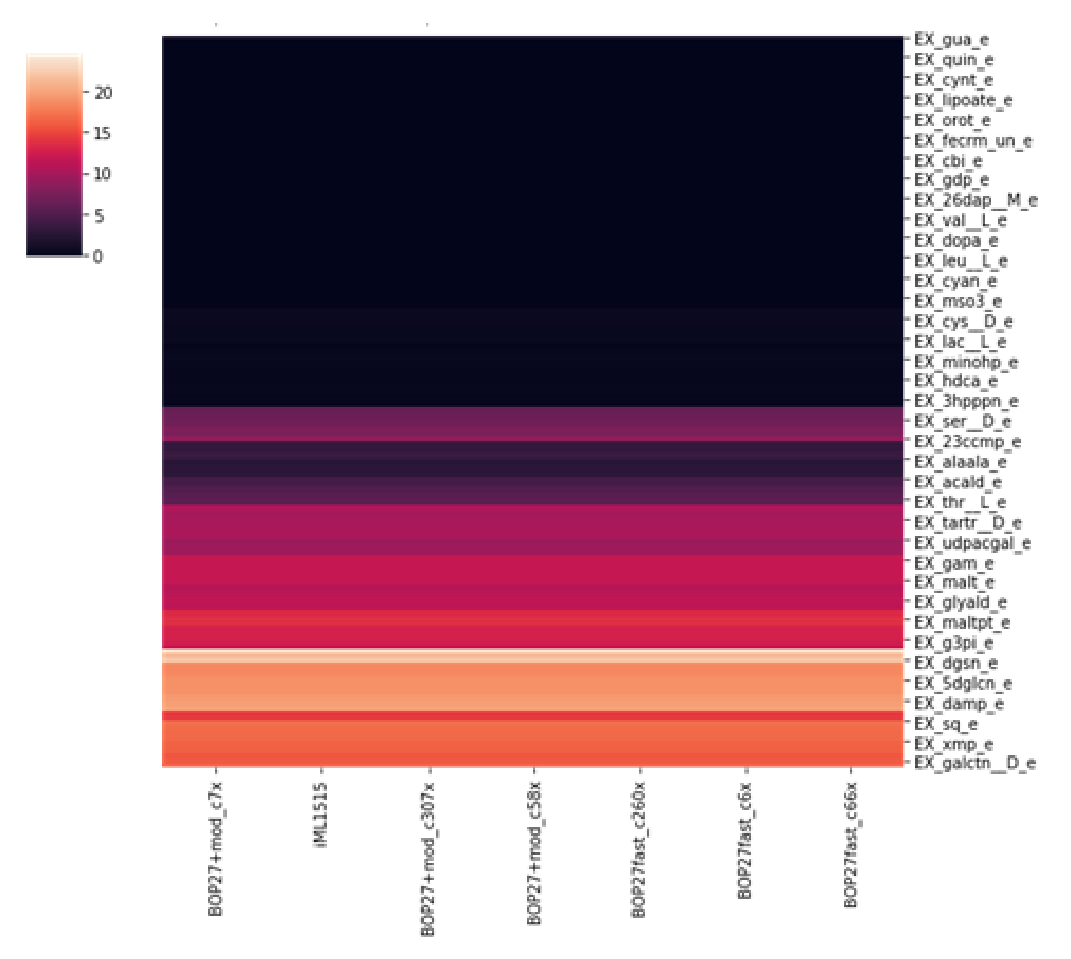


**Supplementary Figure 2.** Growth rate simulations for GEMs derived from the assemblies generated from MinION sequencing reads on various carbon sources. BOP27+mod: high accuracy basecalling algorithm with methylation calling, BOP27fast: fast bascalling algorithm, iML1515: reference GEM (Monk et al., 2017).


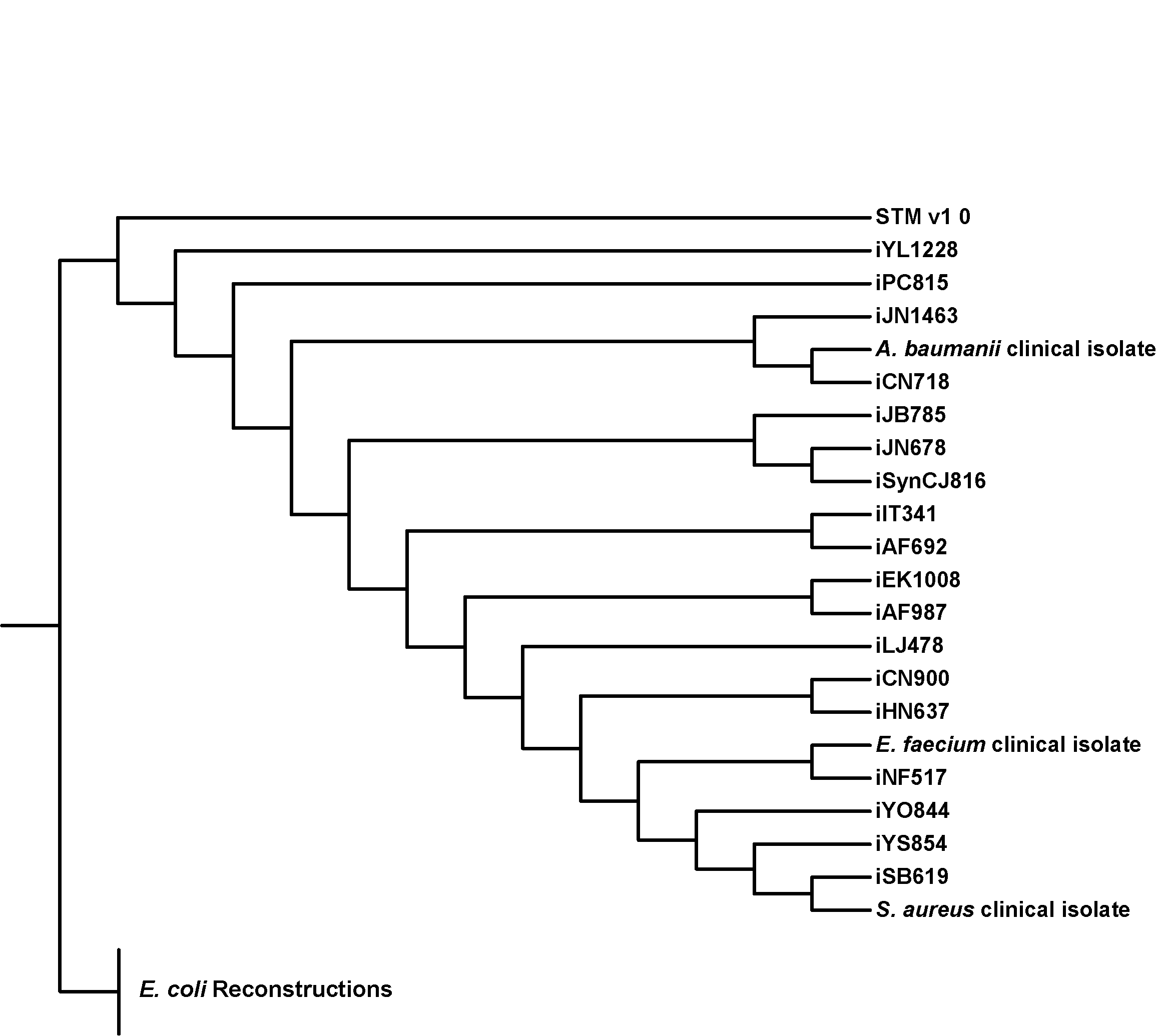


**Supplementary Figure 3.** Phylogenomic analysis of the clinical isolates investigated and the species present in the BiGG Models Database.


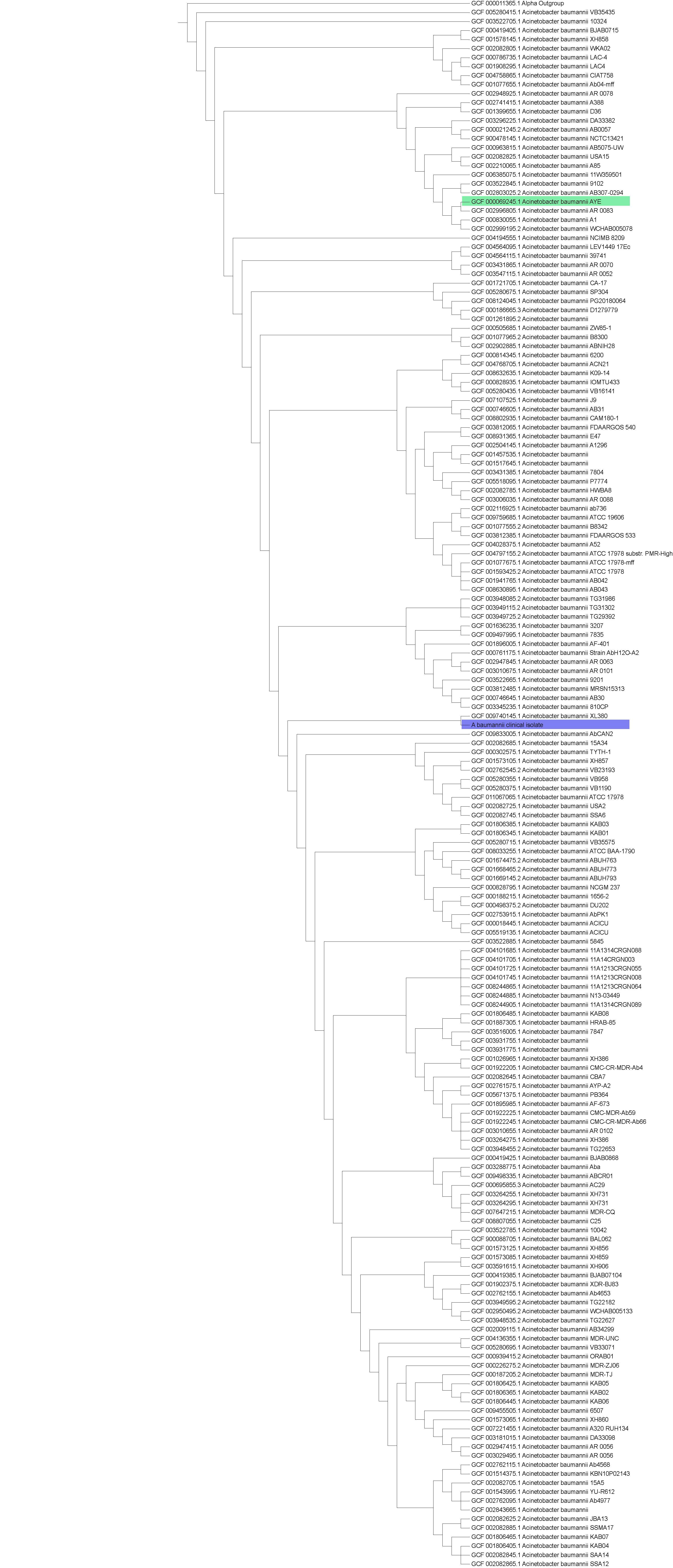


**Supplementary Figure 4.** Phylogenomic analysis of the *A. baumannii* clinical isolate in this investigation and all *A. baumannii* strains in the RefSeq database.

## Supplementary Tables

NCBI accession numbers for GToTree: See included file **Table_S1_ToL_accession_num.xlsx**

**Supplementary Table 2:** Effect of sequence coverage depth on the assembly of E. coli strain K12 substrain BOP27.

|  | **Coverage** | **Contigs** | **Q score** | **SNPs** | **InDels** | **Assembly time (min)** | **CDS** | **tRNA** | **rRNA** |
| --- | --- | --- | --- | --- | --- | --- | --- | --- | --- |
| **HAC+** | **7x** | **5** | **22.5** | **4816** | **18918** | **8** | **7881** | **86** | **22** |
|  | **11x** | **1** | **25.6** | **1178** | **8598** | **8** | **6613** | **85** | **22** |
|  | **14x** | **1** | **27.2** | **359** | **5017** | **10** | **5936** | **87** | **22** |
|  | **27x** | **1** | **29.3** | **104** | **2284** | **17** | **5016** | **88** | **22** |
|  | **58x** | **1** | **30.4** | **33** | **1117** | **31** | **4694** | **88** | **22** |
|  | **307x** | **1** | **32.0** | **21** | **598** | **208** | **4488** | **88** | **22** |
| **K12 ref** | **N/A** | **N/A** | **N/A** | **N/A** | **N/A** | **N/A** | **4305** | **88** | **22** |

**Supplementary Table 3.** Demultiplexed barcoded samples from the mock metagenome.

| **Barcodes** | **Reads** | **% reads** | **% nts** | **ID** |
| --- | --- | --- | --- | --- |
| **BC03** | **4570** | **4%** | **1%** | ***S. elongatus* PCC 7942 carry-over** |
| **BC07** | **7297** | **6%** | **8%** | ***A. baumannii***  **1 plasmid** |
| **BC08** | **13376** | **12%** | **3%** | ***E. coli* CFT073** |
| **BC09** | **19543** | **17%** | **26%** | ***E. faecium***  **4 plasmids** |
| **BC11** | **35428** | **30%** | **30%** | ***S. aureus*** |
| **BC12** | **23429** | **20%** | **18%** | ***E. coli* O157:H7**  **1 plasmid** |
| **None** | **12507** | **11%** | **13%** |  |

**Supplementary Table 4.** Assembly statistics for *E. coli* O157:H7 demultiplexed reads.

| **#seq_name** | **length** | **cov.** | **circ.** |
| --- | --- | --- | --- |
| **contig_1** | **225662** | **13** | **-** |
| **contig_2** | **239902** | **14** | **-** |
| **contig_3** | **59183** | **30** | **-** |
| **contig_4** | **4330067** | **22** | **-** |
| **contig_5** | **324857** | **19** | **-** |
| **contig_10** | **116674** | **15** | **-** |
| **contig_11** | **92278** | **42** | **+** |
| **contig_14** | **158821** | **12** | **-** |
| **contig_15** | **10590** | **21** | **-** |
| **contig_17** | **16589** | **62** | **+** |

**References (all Supplementary References also appear in the Main Text)**

Ebrahim, A., Lerman, J. A., Palsson, B. O., and Hyduke, D. R. (2013). COBRApy: COnstraints-Based Reconstruction and Analysis for Python. *BMC Systems Biology* 7, 74. doi:10.1186/1752-0509-7-74.

King, Z. A., Lu, J., Dräger, A., Miller, P., Federowicz, S., Lerman, J. A., et al. (2016). BiGG Models: A platform for integrating, standardizing and sharing genome-scale models. *Nucleic Acids Res* 44, D515–D522. doi:10.1093/nar/gkv1049.

Monk, J. M., Lloyd, C. J., Brunk, E., Mih, N., Sastry, A., King, Z., et al. (2017). iML1515, a knowledgebase that computes Escherichia coli traits. *Nat Biotechnol* 35, 904–908. doi:10.1038/nbt.3956.

Norsigian, C. J., Pusarla, N., McConn, J. L., Yurkovich, J. T., Dräger, A., Palsson, B. O., et al. (2020). BiGG Models 2020: multi-strain genome-scale models and expansion across the phylogenetic tree. *Nucleic Acids Res* 48, D402–D406. doi:10.1093/nar/gkz1054.
